# Optimistic people are all alike: Shared neural representations supporting episodic future thinking among optimistic individuals

**DOI:** 10.1073/pnas.2511101122

**Published:** 2025-07-21

**Authors:** Kuniaki Yanagisawa, Ryusuke Nakai, Kohei Asano, Emiko S. Kashima, Hitomi Sugiura, Nobuhito Abe

**Affiliations:** ^a^Department of Psychology, Graduate School of Humanities, Kobe University, Nada-Ku, Kobe 657-8501, Japan; ^b^Institute for the Future of Human Society, Kyoto University, Yoshida Sakyo-Ku, Kyoto 606-8501, Japan; ^c^Department of Child Care and Education, Faculty of Child Care and Education, Osaka University of Comprehensive Children Education, Higashisumiyoshi-Ku, Osaka 546-0021, Japan; ^d^Department of Psychology, Counselling and Theory, School of Psychology and Public Health, La Trobe University, Bundoora, VIC 3086, Australia; ^e^Department of Career Management, Faculty of Business Administration, Kindai University, Higashiosaka City, Osaka 577-8502, Japan

**Keywords:** optimism, Anna Karenina principle, MPFC, INDSCAL, IS-RSA

## Abstract

Optimism is a critical personality trait that influences future-oriented cognition by emphasizing positive future outcomes and deemphasizing negative outcomes. How does the brain represent idiosyncratic differences in episodic future thinking that are modulated by optimism? In two functional MRI (fMRI) studies, participants were scanned during an episodic future thinking task in which they were presented with a series of episodic scenarios with different emotional valence and prompted to imagine themself (or their partner) in the situation. Intersubject representational similarity analysis revealed that more optimistic individuals had similar neural representations in the medial prefrontal cortex (MPFC), while less optimistic individuals exhibited more idiosyncratic neural representations in the MPFC. Additionally, individual difference multidimensional scaling of MPFC activity revealed that the referential target and emotional valence of imagined events were clearly mapped onto different dimensions. Notably, the weights along the emotional dimension were closely linked to the optimism scores of participants, suggesting that optimistic individuals imagine positive events as more distinct from negative events. These results suggest that shared neural processing of the MPFC among optimistic individuals supports episodic future thinking that facilitates psychological differentiation between positive and negative future events.

## Significance

Optimism, defined as maintaining positive expectations for the future, is a crucial psychological resource correlated with enhanced well-being and physical health. Recent research suggests that neural processing of cognitive function is similar among individuals with positive traits but more dissimilar among those with negative traits. Applying the cross-subject neural representational analytical approach, we found that optimistic individuals display similar neural processing when imagining the future, whereas less optimistic individuals show idiosyncratic differences. Additionally, we found that optimistic individuals imagined positive events as more distinct from negative events than less optimistic individuals. These results have both theoretical and methodological implications for our understanding of the adaptive nature of optimism.

Optimism, characterized by a positive outlook on the future, plays a vital role in shaping one’s cognitive structure and influences how we perceive various future scenarios. For example, optimistic individuals envision positive future events not only more vividly than negative events but also as are more likely to happen soon, as if they were experiencing them in the present moment (1, 2). Such an optimistic cognitive structure can buffer against negative mood (3) and mitigate stress (4). More generally, optimism has a positive impact not only on general well-being but also on mental and physical health (5).

Despite the remarkable benefits of optimism on one’s health and well-being and the impact of optimism on episodic future thinking in which positive future outcomes are emphasized and negative outcomes are deemphasized, it remains unclear how the brain represents these optimism-modulated idiosyncratic differences in episodic future thinking. Recent neuroimaging studies indicate that individuals who share a positive trait, such as a higher social network position, exhibit convergent neural responses in the medial prefrontal cortex (MPFC) (6). Based on these findings, we hypothesized that optimism, another well-documented positive trait, would similarly promote neural convergence in the MPFC. Indeed, research suggests that highly optimistic individuals often maintain broader social networks (7), implying greater alignment in anticipating and engaging with future events. Furthermore, Iliev and Bennis (8) reported that individuals high in multiple positive traits (e.g., optimism, self-esteem, and life satisfaction) tend to share similar personality dimensions, suggesting that positivity fosters shared ways of interpreting experiences. By emphasizing positive scenarios and downplaying negative ones, optimism may lead to more uniform patterns of future-oriented neural activity, particularly in the MPFC—a region closely associated with self-referential and prospective thinking. We drew on the Anna Karenina principle—which is derived from the famous opening line of Leo Tolstoy’s novel Anna Karenina (i.e., “Happy families are all alike; every unhappy family is unhappy in its own way”) and posits that successful outcomes exhibit similar characteristics whereas unsuccessful outcomes vary widely—to hypothesize that highly optimistic individuals exhibit more similar neural representations, whereas less optimistic individuals exhibit more idiosyncratic patterns. This hypothesis is in line with previously reported evidence that has suggested that positive traits foster neural convergence, whereas negative traits—such as loneliness—lead to increased variability in neural responses (9). On the basis of this principle, we propose that “optimistic individuals are all alike, but each less optimistic individual imagines the future in their own way.”

To assess the neural similarity during the imagining of future scenarios across participants, we conducted two fMRI studies involving healthy adults performing an episodic future thinking task. Participants were presented with a series of episodic scenarios with different emotional valences (positive, neutral, negative, and death-related) that prompted them to imagine their (or their partner’s) future. The task included self-referential and partner-referential conditions to capture a broad range of cognitive and emotional contexts. This design allowed us to investigate whether optimism-related neural convergence occurs consistently across self- and other-focused perspectives or is specific to imagining one’s own future. In Study 1, we included death-related scenarios to examine responses to extremely negative future events. However, this approach created an imbalance in the distribution of negative emotional conditions. Study 2 excluded the death condition to address this issue, ensuring a more balanced design with positive, neutral, and negative scenarios. These experimental conditions were selected to elicit meaningful variability in episodic future thinking task across participants, making them well-suited for intersubject representational similarity analysis (IS-RSA) (10–12).

We applied IS-RSA by calculating the dissimilarity of activity patterns obtained from multivariate pattern analysis (MVPA) between every pair of participants (13–16), which were used to compare with two behavioral dissimilarity matrices based on participants’ optimism scores. The first candidate model, the nearest neighbor (NN) model, states that participants with similar optimism scores will have similar neural representations, regardless of whether those scores are high or low. In this model, the dissimilarity matrix is based only on the absolute value of the difference between each pair of participants in the optimism scale. The second model, i.e., the Anna Karenina (AnnaK) model, posits that only pairs featuring two optimistic individuals exhibit very similar neural representations of episodic future thinking. In this model, we first ranked participants on the basis of their optimism scores and then computed the normalized average rank for each pair. The dissimilarity between each pair was defined as one minus this normalized average rank, resulting in smaller dissimilarity values (i.e., higher levels of similarity) for pairs of highly optimistic individuals. We investigated whether the predictions made by the AnnaK model were more closely associated with the neural dissimilarity matrices than were those made by the NN model.

Our IS-RSA focused on the default mode network (DMN), particularly the MPFC. The DMN (17), comprising regions in the MPFC, medial temporal lobe, posterior cingulate cortex/precuneus, and lateral temporal and parietal regions, corresponds to the episodic future thinking network and functions adaptively to integrate information about relationships and associations from past experiences to construct mental simulations about possible future events (18–21). The DMN broadly supports episodic future thinking; however, we prioritized the MPFC due to its unique role in integrating emotional and self-referential processes, which is possibly critical for optimism-driven neural convergence. Prior studies using MVPA have demonstrated that the neural representations in the MPFC can carry information about individuals (22) and emotions (23) while imagining episodic events. We therefore expect that our IS-RSA can capture the idiosyncratic differences in neural representations of the MPFC that reflect the person’s simulated emotional experience, with individual differences in optimism as a modulating factor.

While IS-RSA can effectively elucidate any intersubject variability in episodic future thinking modulated by optimism, it does not reveal the exact underlying cognitive structures represented in the MPFC. To compensate for this limitation, we conducted an individual difference multidimensional scaling (INDSCAL) model to further identify the lower-dimensional representation from the patterns of brain activity in the MPFC across the participants. This approach enabled us to explore whether the neural representations associated with imagining positive and negative future events differ in emotional dimensions. Additionally, INDSCAL calculates weights for each subject along each of the dimensions, signifying the importance of each dimension for each subject (24, 25). Hence, the magnitude of weights in the emotional dimension can provide valuable insights into how individuals perceive future positive events differently from negative ones.

Here, using IS-RSA and INDSCAL across two fMRI experiments, we show that shared neural processing in the MPFC among optimistic individuals supports episodic future thinking that facilitates psychological differentiation between positive and negative future events. We also propose that this combination of IS-RSA and INDSCAL will be instrumental in allowing cognitive neuroscience research to shift focus from the group to the individual because this combined analysis can reveal subtle idiosyncratic differences in patterns of brain activity that cannot be captured by conventional analysis while at the same time visualizing and revealing the underlying cognitive structures.

## Results

### Associations between Optimism and Neural Similarity.

**Fig. 1.**
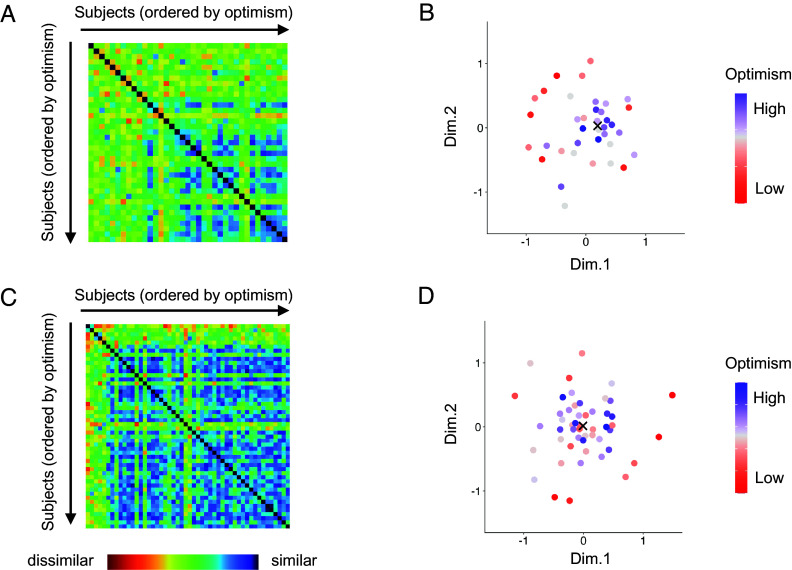
(*A*) Associations between intersubject dissimilarity and optimism in Study 1. This matrix pertains to an ROI in the MPFC, with the rows and columns arranged in ascending order of optimism. The *Top Left* corner represents the pair with the lowest optimism scores, while the *Bottom Right* corner corresponds to the pair with the highest optimism scores. Warm colors represent high dissimilarity values, while cool colors represent low dissimilarity values. (*B*) MDS visualization for Study 1. Nonmetric MDS was applied to represent the degree of similarity among participants based on their neural patterns in the MPFC. Each dot represents a participant, and colors reflect optimism scores. Gaussian kernel density estimation was used to identify the region with the highest density of points, which is marked with a cross. (*C*) Associations between intersubject dissimilarity and optimism in Study 2. The matrix was constructed using the same procedure as in Study 1. (*D*) MDS results for Study 2. MDS was applied using the same procedure as in Study 1, and the densest region in the MDS space was identified and marked with a cross. Note that Dimension 1 and Dimension 2 of MDS presented here are different from Dimension 1 and Dimension 2 in the INDSCAL results presented later.

We developed participant-by-participant neural dissimilarity matrices for Studies 1 and 2 by ordering participants according to their optimism scores (see Fig. 1 *A* and *C* for MPFC) and examining whether intersubject neural representational similarity in DMN regions of interest (ROIs) was associated with optimism scores. Visual inspection of these matrices suggests a relatively continuous increase in neural representational similarities along the diagonal in both studies, indicating that pairs consisting of two optimistic individuals have very similar neural representations of episodic future thinking. Based on these matrices, we also conducted multidimensional scaling (MDS) to visualize the distance between participants in a two-dimensional space (Fig. 1 *B* and *D*). Each dot represents a subject, and subjects are colored according to their optimism scores. Consistent across both studies, MDS results also suggest that optimistic individuals are closer to each other (i.e., convergence of optimism), while less optimistic individuals are farther apart. To quantify these relationships, we identified the densest region in the MDS space for each study (marked with a cross in Fig. 1 *B* and *D*), and calculated each participant’s distance from this region. Spearman correlation analyses revealed significant negative correlations between distance from the densest region and optimism scores in both Study 1 (ρ = −0.63, *P* < 0.001) and Study 2 (ρ = −0.58, *P* < 0.001), indicating that higher optimism scores were associated with increased proximity to the densest region. These findings suggest that optimistic individuals consistently exhibit convergent neural representations, reflecting shared patterns of episodic future thinking, whereas less optimistic individuals demonstrate increased variability across studies.

To further interpret these results, we then conducted the IS-RSA in DMN ROIs for Studies 1 and 2. This analysis tested whether individuals with similar optimism scores have very similar neural representations of episodic future thinking using a dissimilarity matrix based on the NN model. We observed no significant correlations between the true neural dissimilarity matrix and the NN model for any ROI (*Ps* > 0.05) across both studies.

We next conducted the IS-RSA to determine whether only pairs with two optimistic individuals show very similar neural representations of episodic future thinking, using a dissimilarity matrix that reflect the predictions of the AnnaK model. In Study 1, we observed a significant correlation between the true neural dissimilarity matrix and the AnnaK modeled matrix in four ROIs, including the MPFC (ρ = 0.42, Bonferroni-corrected *P* = 0.001), left parahippocampal gyrus (ρ = 0.13, Bonferroni-corrected *P* = 0.003), left middle temporal gyrus (ρ = 0.34, Bonferroni-corrected *P* = 0.012), and right superior frontal gyrus (ρ = 0.32, Bonferroni-corrected *P* = 0.009). In Study 2, we observed significant correlations in six ROIs, including the MPFC (ρ = 0.38, Bonferroni-corrected *P* = 0.003), precuneus (ρ = 0.29, Bonferroni-corrected *P* = 0.024), cerebellum (ρ = 0.16, Bonferroni-corrected *P* = 0.041), left middle temporal gyrus (ρ = 0.23, Bonferroni-corrected *P* = 0.034), right angular gyrus (ρ = 0.28, Bonferroni-corrected *P* = 0.036), and right superior frontal gyrus (ρ = 0.31, Bonferroni-corrected *P* = 0.016). Detailed correlations between the neural and model RDMs for all the ROIs, including correlation coefficients and Bonferroni-corrected *P* values, are presented in full in *SI Appendix*, Table S1. Moreover, the results regarding the MPFC and right superior frontal gyrus remained significant after controlling for the NN model and additional control variables in Study 2 (*SI Appendix*, *Supplementary Results*). Additional analysis using representational similarity measures at the dyadic level also yielded broadly consistent results across both studies (*SI Appendix*, *Supplementary Results*). These results indicate that pairs in which both individuals were optimistic were characterized by higher neural similarity than pairs in which one or both individuals were less optimistic.

To further investigate whether the observed patterns differ between self-referential and partner-referential contexts, we conducted condition-specific IS-RSA in Study 1. In the self-condition, the MPFC showed a significant correlation with the AnnaK model (ρ = 0.26, Bonferroni-corrected *P* = 0.019), consistent with the primary findings that included all conditions. No significant correlations were observed for other ROIs with the AnnaK or NN models (Bonferroni-corrected *P*s > 0.050). In the partner condition, while the MPFC did not reach statistical significance with the AnnaK model (ρ = 0.26, Bonferroni-corrected *P* = 0.051), a similar trend was observed. None of the other ROIs showed significant correlations with the AnnaK or NN models (Bonferroni-corrected *P*s > 0.050). In light of previous evidence indicating that optimism biases often extend to close others (e.g., ref. 26), this marginal result in the partner condition likely reflects limited statistical power rather than the genuine absence of an effect, thus necessitating further investigation. Condition-specific analyses were not conducted in Study 2 given that this study featured an insufficient number of conditions (i.e., a 3-by-3 RDM, resulting in only 3 unique cells).

To determine whether univariate differences in overall activity levels within each ROI could explain the representational patterns identified by IS-RSA, we conducted additional analyses with the assistance of Euclidean distance matrices that were derived from univariate signal changes to represent pairwise dissimilarities between conditions. We performed IS-RSA by comparing these univariate-based matrices with the AnnaK and NN models on the basis of Mantel tests (Bonferroni-corrected across 11 ROIs). In Study 1, no significant correlations were observed between the univariate-based distance matrices and either the AnnaK or NN models in any ROI (Bonferroni-corrected *P*s > 0.050). Similarly, in Study 2, no significant correlations emerged with respect to the AnnaK model (Bonferroni-corrected *P*s > 0.050), although significant correlations were observed with the NN model in three ROIs (precuneus: ρ = 0.19, Bonferroni-corrected *P* = 0.031; right middle temporal gyrus: ρ = 0.20, Bonferroni-corrected *P* = 0.047; left middle temporal gyrus: ρ = 0.19, Bonferroni-corrected *P* = 0.038). Additionally, correlations between univariate-based and MVPA-based matrices were consistently low and nonsignificant across all the ROIs in both studies (ρ range: –0.079 to 0.145, all Bonferroni-corrected *P*s > 0.050). These findings indicate that univariate effects alone cannot adequately account for the representational patterns identified by the multivariate analyses. The detailed methods and complete results associated with each study and ROI are provided in *SI Appendix*.

### Lower-Dimensional Representation of Neural Activities in the MPFC.

**Fig. 2.**
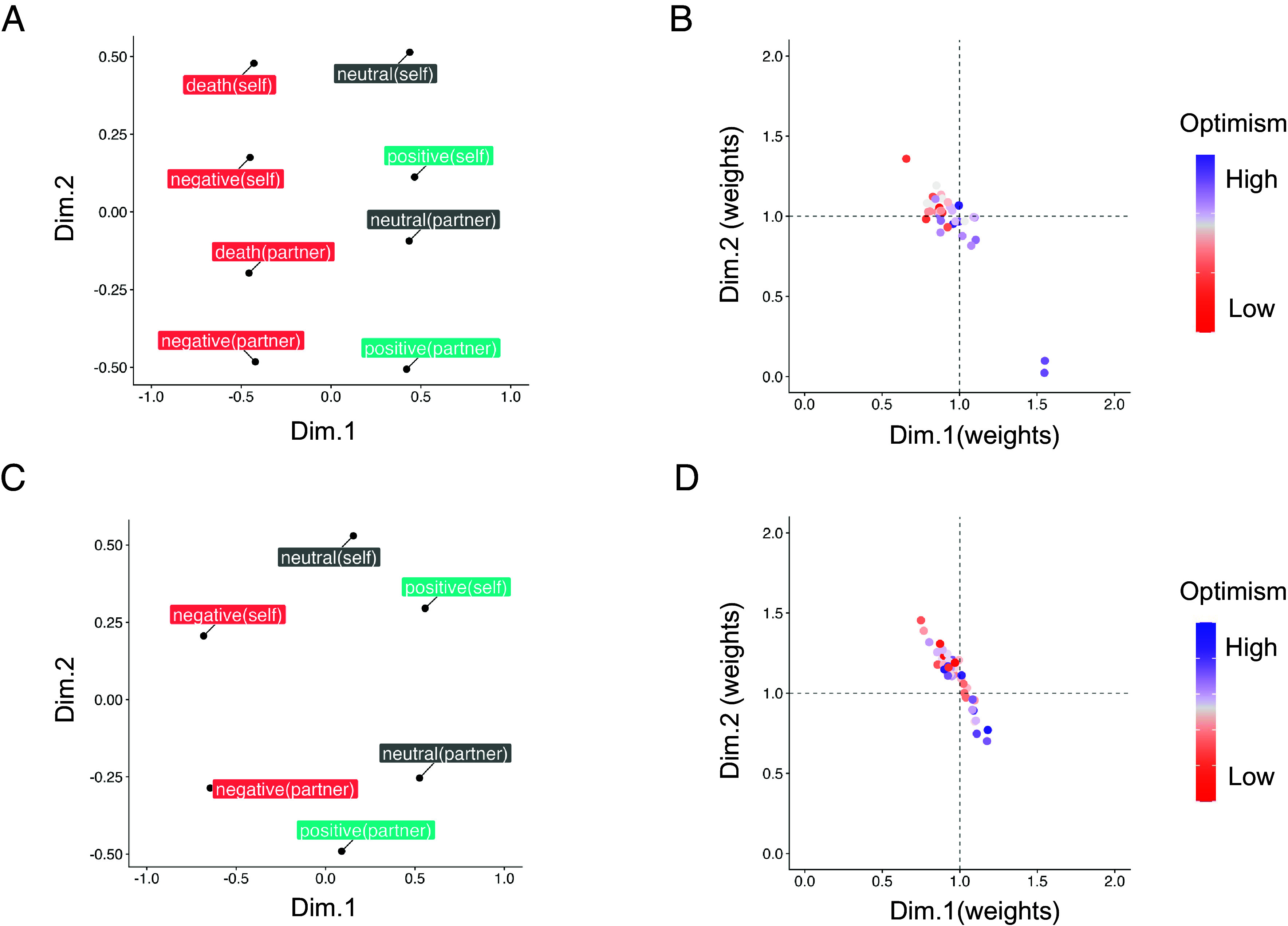
(*A*) Lower-dimensional representation of future-thinking space in the MPFC ROI based on fMRI data using the INDSCAL approach for Study 1. Each color represents an emotional valence. (*B*) Individual subject weights for Study 1, indicating the importance of the two dimensions for each participant. Each dot represents a participant, with colors reflecting optimism scores. (*C*) Lower-dimensional representation for Study 2 was derived using the same procedure as Study 1. (*D*) Individual subject weights for Study 2 were also derived using the same procedures as Study 1.

To investigate the lower-dimensional representation of neural activities in the MPFC, we extracted the neural RDM from the MPFC and conducted an INDSCAL analysis for Studies 1 and 2. In study 1, all 8 × 8 correlation matrices of participant neural representations were input into the INDSCAL models, and a common configuration was abstracted with fit indices. INDSCAL models with 2, 3, or 4 dimensions were fitted to the data, and the resulting stress values were 0.25, 0.15, and 0.11, respectively. The INDSCAL model with three dimensions showed good fit metrics, and adding a fourth dimension did not provide easily interpretable information. Therefore, models with three dimensions were chosen as suitable representations of the fMRI data. For simplicity, we focus on the first and second dimensions for the MPFC ROI, whereas we have described the third dimension in *SI Appendix*, Fig. S1. In Study 2, 6 × 6 correlation matrices were used as input for the INDSCAL analysis, with stress values of 0.19, and 0.10 for the two- and three-dimensional models, respectively. The two-dimensional model was selected as it provided a balance between fit and interpretability. The common two-dimensional space illustrated in Fig. 2 *A* and *C* shows that the first dimension reflected valence, separating positive and negative affective trials. In contrast, the second dimension reflected the referential target, distinguishing between self- and partner-referential trials. As shown in *SI Appendix*, in Study 1, the scores along Dimension 1 were highly correlated with the valence scores of experimental conditions rated by an independent sample for stimulus validation (*SI Appendix*) (ρ = −0.81, *P* = 0.011, one-tailed), and the scores along Dimension 2 were highly correlated with the referential target condition (ρ = −0.87, *P* = 0.002, one-tailed). A similar pattern was observed in Study 2, in which Dimension 1 reflected valence (ρ = −0.77, *P* = 0.051, one-tailed) and Dimension 2 reflected the referential target condition (ρ = −0.88, *P* = 0.011, one-tailed).

The INDSCAL model also provides weights for individual subjects along the two dimensions, and a plot of these weights is shown in Fig. 2 *B* and *D*. Weights are used to gain insight into each subject’s internal visualization space; they indicate the importance of the two dimensions, which are idiosyncratic for individual subjects. Those with low optimism scores tended to fall within the second quadrant of the model, while those with high optimism scores tended to fall within the fourth quadrant. Consistent with this result, there was a significant positive correlation between optimism scores and the weight of Dimension 1 (ρ = 0.69, *P* < 0.001) in Study 1, indicating that optimistic individuals placed greater emphasis on valence differentiation. Similar results were observed in Study 2 (ρ = 0.30, *P* < 0.05). In addition, there was a significant negative correlation between optimism scores and the weight of Dimension 2 (ρ = −0.44, *P* = 0.006) in Study 1, indicating that optimistic individuals placed less emphasis on self-other differentiation. However, this relationship was not statistically significant in Study 2 (ρ = −0.24, *P* = 0.100). These results show that the model solution for optimistic individuals would be similar to that in Fig. 2*A* (Study 1) and 2*C* (Study 2) but stretched out along the first dimension in an elliptical fashion. Therefore, our neural data supported the idea that optimistic individuals imagined positive events as more distant from negative ones.

## Discussion

We used fMRI and an episodic future thinking task to test the prediction that neural processing of imagining future events is similar in highly optimistic individuals but dissimilar in less optimistic individuals. As predicted, IS-RSA revealed that more optimistic individuals had similar neural representations in the MPFC, whereas less optimistic individuals exhibited more idiosyncratic neural representations in the MPFC. Performing INDSCAL on neural data of the MPFC revealed that the referential target and emotional valence of imagined events are clearly mapped on different dimensions. Furthermore, the weights along the emotional dimensions were positively correlated with optimism scores across participants. These findings were highly replicable across Study 1 and Study 2 despite differences in task design.

These findings illuminate how optimistic individuals imagine future events in two characteristic ways. First, they link episodic future thinking among highly optimistic individuals to shared neural processing of the MPFC. These results are in line with a priori prediction derived from the Anna Karenina principle: “Optimistic individuals are all alike, but each less-optimistic individual imagine the future in their own way.” Notably, the AnnaK model supported this pattern more robustly than simpler models such as the NN approach. The results are also consistent with a recent fMRI study showing that people who share some positive individual-difference trait (more specifically, higher social network position; see ref. 6) show similar neural responses. Furthermore, our findings support the broader “convergence of positivity” hypothesis, which suggests that individuals with high levels of positive traits, such as optimism, share specific psychological and neural characteristics (8). Extending this hypothesis to the neural level, our findings demonstrate shared MPFC representations among optimistic individuals, highlighting a “neural convergence of optimism.”

Second, these findings support the idea that optimistic individuals imagine the future in ways that enhance psychological differentiation between positive and negative events. Using INDSCAL, we extracted neural representations in the MPFC along two clear dimensions: emotional valence and referential context (self vs. partner). Consistent with our hypotheses, optimistic individuals showed greater differentiation between positive and negative events along the emotional dimension. This suggests a cognitive structure in which optimistic individuals distinctly prioritize positive events over negative ones. In contrast, the relationship between optimism and differentiation along the self vs. partner dimension was less clear; an exploratory analysis revealed that optimistic individuals placed less emphasis on self-other differentiation in Study 1, but this finding was not replicated in Study 2. Thus, while a potential link between optimism and decreased self-other differentiation is intriguing, this finding should be interpreted cautiously.

At first glance, the greater neural separation between positive and negative events observed among optimistic individuals might seem to be counterintuitive. One might assume that optimistic individuals would minimize the psychological distance between positive and negative events by reinterpreting negative scenarios in a more positive or neutral light. However, previous research (1, 2) has indicated that optimism does not primarily involve positive reinterpretations of negative events. Instead, optimistic individuals typically process negative future scenarios in a more abstract and psychologically distant manner, thus mitigating the emotional impact of such scenarios. By contrast, when envisioning positive events, optimistic individuals imagine them vividly and concretely, thus reinforcing the emotional significance and clarity of these scenarios. This asymmetric cognitive-emotional processing effectively enhances the neural differentiation between positive and negative events, which is consistent with the INDSCAL results observed in this research.

To ensure that the observed MPFC effects were not solely driven by differences in activity levels associated with emotional valence, we conducted additional analyses using univariate-based matrices in IS-RSA. These analyses revealed no significant relationship between optimism and MPFC activity, indicating that differences in activity levels related to emotional valence do not fully account for the observed IS-RSA patterns. This interpretation aligns with the findings of Sharot et al. (2), which reported no significant relationship between optimism and subjective ratings of emotional valence for imagined positive or negative events. The present results and previous findings suggest that our main findings illuminate mechanisms beyond those captured by parametric modulation of emotional valence.

From a methodological perspective, we argue that the combination of INDSCAL and IS-RSA is highly appropriate and promising for analyzing data from an individual-difference perspective, as it enables us to assess the similarity of the neural representations across participants and further identify the underlying cognitive structures. Many studies of MVPA have focused solely on one aspect, either assessing the similarities of neural representations among participants (6, 9) or examining cognitive structures (27–29). While use of IS-RSA is well aligned with the recent trend of emphasizing individual differences in cognitive neuroscience (30), it provides limited evidence reflecting the exact nature of the underlying cognitive structures. INDSCAL can compensate for this limitation by visualizing and revealing the cognitive structures at lower dimensions. Therefore, we propose that our complementary approach of using both IS-RSA and INDSCAL has the potential to reveal subtle and idiosyncratic, but interpretable and thus meaningful individual differences above and beyond those revealed through the conventional analytic approach.

We speculate that the observed shared cognitive structure positively impacts social relationships. Previous studies have demonstrated that, relative to pessimism, optimism is linked to larger social networks, higher relationship satisfaction, and increased perceived social support (31, 32). Moreover, optimists are perceived as more likable and attractive than pessimists (33) and more likely to be accepted by their peer group (34). The impact of optimism on social interactions may be rooted in an individual’s optimistic personality traits, but it may also arise from shared cognitive structures among individuals. Indeed, a recent neuroimaging study has shown that the neural responses of individuals who occupy central positions in their social networks are very similar to those of their peers; this similarity is especially evident in regions of the DMN where similar responses have been associated with shared perspectives and subjective understanding (6). Hence, the observed variations in cognitive structure between highly optimistic and less optimistic individuals in our study may play a significant role in enhancing social connection.

This study has one major limitation. Optimism-related neural convergence was clearly observed in the context of self-referential future thinking, although the results pertaining to the partner-referential context were less robust. Previous psychological studies have suggested that optimism often extends beyond the self to influence how individuals perceive the futures of close others (e.g., ref. 26). In light of this observation, we interpret the absence of a statistically significant effect in the partner condition cautiously, given that it is likely the result of methodological constraints rather than a genuine context-specific limitation. Future researchers can employ designs that are optimized for examining partner-focused scenarios to clarify how broadly optimism-driven neural convergence generalizes across self- and partner-referential contexts in further detail.

In summary, optimistic individuals exhibit similar neural processing when they imagine the future, whereas less optimistic individuals exhibit idiosyncratic differences. We also observed that optimistic individuals imagine positive events as more distinct from negative events than do less optimistic individuals. Overall, our research findings, which are derived from a combination of IS-RSA and INDSCAL, suggest the existence of shared neurocognitive representations based on the emotional dimension among optimistic individuals, despite the fact that different individuals may perceive the same future event differently.

## Materials and Methods

### Participants.

For Study 1, we recruited 37 healthy right-handed participants (17 males and 20 females; age range: 28 to 44 y, Mean = 37.03 y) with no history of neurological or psychiatric diseases. For Study 2, we used the same criteria and recruited fifty participants (22 males and 28 females; age range: 25 to 44 y, Mean = 34.02 y). All participants in both studies were married. We determined the sample sizes based on previous fMRI studies using similar paradigms (23) and IS-RSA methods (35, 36). Additionally, recent studies with similar objectives have employed comparable sample sizes, further supporting our selection (37). This study was approved by the Ethics Committee of Kyoto University, and written informed consent was obtained from all participants.

### Optimism Score.

All participants completed a questionnaire after the MRI scanning session. In Study 1, we administered Revised Life Orientation Test (LOT-R) (38, 39) to assess optimism levels. The scale consists of 10 items: three measuring optimism, three measuring pessimism (reverse score item), and four filler items. The response scale ranged from 1 (“strongly disagree”) to 5 (“strongly agree”) (ω = 0.72), and the scores for each statement were summed. In Study 2, in addition to completing the LOT-R (ω = 0.78), participants provided information on their education level, completed Raven’s Coloured Progressive Matrices (RCPM) (40, 41) as a measure of nonverbal intelligence, and reported their socioeconomic status (42, 43). We included these additional measures to assess the potential influence of intelligence and demographic factors on the primary findings.

### Stimuli.

All stimuli were presented using Presentation software (Neurobehavioral Systems, USA). In Study 1, the stimuli consisted of 80 short descriptions of possible life events, including 20 descriptions of positive events (e.g., “You will take an epic trip around the world”), 20 descriptions of neutral events (e.g., “You will submit your resume”), 20 descriptions of negative events (e.g., “You will be fired by your company”), and 20 descriptions of death-related events (e.g., “You will be diagnosed with terminal cancer”), similar to previous studies (23). In Study 2, the stimuli consisted of 60 short descriptions, excluding the 20 death-related events. To validate the stimuli, an independent group of 20 individuals who did not participate in the fMRI study rated each event on an 8-point Likert scale in terms of (1) emotional valence (1 = “extremely positive” to 8 = “extremely negative”), (2) arousal (1 = “not arousing” to 8 = “very arousing”), and (3) semantic death relevance (1 = “not related at all” to 8 = “very strongly related”). As shown in *SI Appendix*, Fig. S2, positive events were rated more positively than neutral, negative, and death-related events. The arousal levels of the positive, negative, and death-related events were similar, but they were all higher than those of the neutral events. Death relevance scores were higher for death-related events than for negative, neutral, and positive events.

### Episodic Future-Thinking Task.

**Fig. 3.**
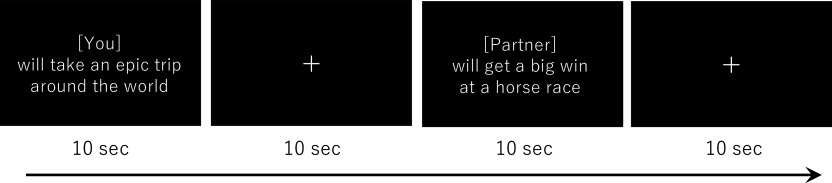
Representative episodic future-thinking task used in the fMRI experiment. In each trial, a description of an event (e.g., “will take an epic trip around the world”) was presented for 10 s.

During the fMRI scans, the participants were instructed to imagine themselves (or their partner) in an event for 10 s with as much detail as possible to experience the situation mentally (Fig. 3). The words “you” or “partner” indicated whether participants should think of the event as occurring to themself or their partner. Participants were not instructed to imagine the events at a specific future temporal point; rather, they were instructed to project each event onto a future temporal point that felt most natural to them. Our separate behavioral experiment revealed that negative events and death-related events were perceived as occurring farther in the future, neutral events as occurring closer in time, and positive events as moderately distant (*SI Appendix*). The instructions presented to participants were as follows:

In this experiment, various events will be presented on the screen. Please pay close attention to the cue word—either “You” or “Partner”—that appears with each event. If the cue is “You,” your task is to vividly imagine the described event happening to yourself in the future. Imagine this scenario for 10 s in as much detail as possible, experiencing it mentally as though it were genuinely happening. If the cue is “Partner,” vividly imagine the described event happening to your partner in the future, again imagining it for 10 s with as much detail as possible, as if your partner were genuinely experiencing it. When you see a “+” sign (fixation) on the screen, simply rest and refrain from thinking about anything in particular until the next event appears.

The fMRI scans consisted of 10 functional runs. In Study 1, each run included 16 trials (two trials for each of four emotional-valence conditions × two referential-target conditions). In contrast, in Study 2, each run included 12 trials (two trials for each of three emotional-valence conditions × two referential-target conditions). We presented the trials in a random order. All the events were presented in each of the self and partner conditions, i.e., once during the first five runs (in the self or partner condition) and once during the second five runs (in the other condition). The interval between each event was 10 s, during which a fixation cross was continuously presented.

After the scanning session, participants performed a surprise recognition task to determine whether they had paid sufficient attention to the stimuli during the fMRI session. In Study 1, participants were shown 80 previously shown and 80 new events, whereas in Study 2, we showed them 60 previously presented and 60 new events. Participants were asked to indicate whether each stimulus was presented during the fMRI scan. We confirmed that participants were attentive to the stimuli and exhibited high recognition performance. In Study 1, the mean hit rate was 90.3% and the mean correct rejection rate was 89.8%. In Study 2, the mean hit rate was 95.1% and the mean correct rejection rate was 96.0%.

### Data Acquisition.

We used the same data acquisition protocol for Studies 1 and 2. Whole-brain imaging was performed using a 3.0-T MAGNETOM Verio MRI scanner (Siemens, Erlangen, Germany). A T2*-weighted echo planar imaging (EPI) sequence sensitive to blood oxygenation level-dependent (BOLD) signals was used for functional imaging with accelerated multiband acquisitions (44–46). The parameters were as follows: repetition time (TR) = 2,000 ms, echo time (TE) = 43 ms, flip angle = 80°, acquisition matrix = 96 × 96, field of view = 192 × 192 mm, in-plane resolution = 2 mm × 2 mm, number of axial slices = 76, multiband factor = 4, and slice thickness without interslice gap = 2 mm (interleaved order). High-resolution (spatial resolution: 1 mm × 1 mm × 1 mm) structural images were acquired using a T1-weighted magnetization-prepared rapid-acquisition gradient echo (MP-RAGE) pulse sequence. Firm padding was placed around each participant’s head to restrict head motion. The visual stimuli were projected onto a screen and viewed through a mirror attached to a 32-channel head coil. The participants’ responses were collected using an MRI-compatible response box. The first five scans in each run were discarded to allow for T1 equilibration effects.

### Data Processing.

We conducted data preprocessing and statistical analysis for Studies 1 and 2 using Statistical Parametric Mapping (SPM)-12 software (Wellcome Department of Imaging Neuroscience, London, UK). All functional images for each participant were corrected for the slice acquisition time. The resulting images were then realigned to correct for small movements that occurred between scans. This process generated an aligned set of images and a mean image for each participant. Each participant’s T1-weighted structural MRI was segmented according to tissue type (gray matter, white matter, and cerebrospinal fluid). Skull-stripped anatomical images were created by combining the segmented images using the ImCalc function in SPM. The expression used was as follows: [(i1 *(i2+i3+i4))>threshold], where i1 was the bias-corrected anatomical scan, and i2, i3, and i4 were the segmented tissue images (gray matter, white matter, and cerebrospinal fluid, respectively). The threshold was set to 0.5 to achieve adequate brain extraction for each participant. Each participant’s realigned EPI images were coregistered to the skull-stripped anatomical image. Using the parameters of the deformation field obtained during segmentation, the EPI images were normalized to the Montreal Neurological Institute (MNI) template. The EPI volumes were spatially smoothed with an isotropic 8-mm full-width at half-maximum (FWHM) Gaussian kernel prior to general linear model (GLM) analysis. Separate GLMs were estimated for the smoothed and unsmoothed data to accommodate different analytical approaches: The smoothed data were utilized for univariate analysis (*SI Appendix*), whereas the unsmoothed data were utilized for MVPA to preserve the maximum amount of spatial information.

Each experimental condition duration (i.e., 10 s) was modeled using a canonical hemodynamic response function. The GLM for Study 1 included eight condition regressors: four emotional-valence conditions (positive, negative, neutral, or death-related) × two referential-target conditions (self or partner). The Study 2 GLM included six condition regressors, as the death-related condition was excluded [three emotional-valence conditions (positive, negative, or neutral) × two referential-target conditions]. The motion parameters (6 regressors for each run) estimated in the realignment procedure were also included in the GLM to regress out potential motion-induced signal fluctuations. A high-pass filter with a cutoff frequency of 1/128 Hz was used to remove low-frequency noise, and a first-order autoregressive [AR(1)] model was employed to correct for temporal autocorrelations. Beta weights from the GLM were converted to t‐statistic images to suppress the contribution of voxels with high beta estimates due to high noise (47).

### Definition of ROIs.

The brain regions that underlie episodic future thinking were defined using a meta-analysis map (association test) of voxels associated with “default mode” from the online database NeuroSynth [http://neurosynth.org (48)]. This ROI (10,715 voxels) comprised brain regions that have been preferentially implicated in neuroimaging studies that addressed the neural bases of the DMN and included areas involved in episodic future thinking. From this mask, we then isolated clusters of individual brain regions that included more than 100 voxels. The clusters included the MPFC (2,416 voxels), precuneus (3,517 voxels), bilateral angular gyri (L, 1,067 voxels; R, 907 voxels), bilateral parahippocampal gyri (L, 154 voxels; R, 112 voxels), bilateral middle temporal gyri (L, 570 voxels; R, 419 voxels), bilateral superior frontal gyri (L, 279 voxels; R, 583 voxels), and cerebellum (130 voxels). Patterns of neural activity in these ROIs were analyzed with the CoSMoMVPA toolbox (49) implemented in MATLAB (MathWorks, Natick, MA). All ROIs are visualized in *SI Appendix*, Fig. S3.

### IS-RSA.

**Fig. 4.**
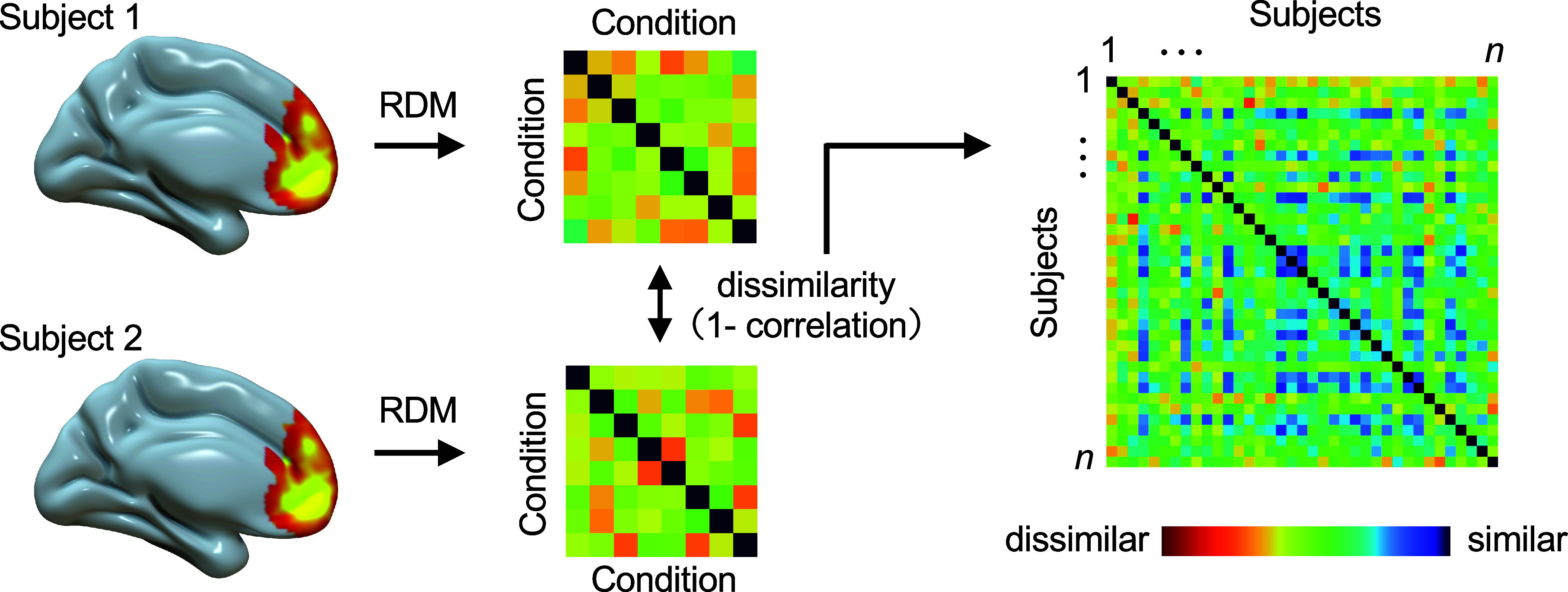
Intersubject RDM. We first performed RSA across all conditions to generate an RDM for each participant in each ROI (*Left*). The resulting RDMs of the participants were then utilized to construct a dissimilarity matrix, employing pairwise correlation to reflect dissimilarity between every pair of participants (*Right*). Brain maps were rendered using the Surf Ice visualization tool (https://www.nitrc.org/projects/surfice/).

IS-RSA was performed using each participant’s neural representational dissimilarity matrix (RDM) within each ROI (Fig. 4). In Study 1, we used an 8 × 8 neural RDM, whereas in Study 2, we employed a 6 × 6 neural RDM to reflect differences in experimental conditions. We developed a dissimilarity matrix for each ROI by calculating the pairwise Spearman correlation dissimilarity between each pair of participants. These distances were transformed into a participant-by-participant dissimilarity matrix and then projected into a two-dimensional space using nonmetric MDS with ordinal scaling. To examine the relationship between optimism and participant distribution in this space, we used Gaussian kernel density estimation to identify the highest-density region in each study, following a previous research (37). We calculated the Euclidean distance of each participant from the densest point to quantify their proximity to the most populated region. This analysis allowed us to determine whether optimistic individuals clustered more closely than less optimistic individuals in the MDS plane.

We also created two other dissimilarity matrices (*SI Appendix*, Fig. S4). First, we constructed a dissimilarity matrix reflecting the NN model based on the absolute value of the difference between each pair of participants in the optimism scale. This matrix allowed us to test the idea that individuals with similar optimism scores have very similar neural representations during episodic future thinking to each other, regardless of whether their objective optimism score is high or low. The matrix normalized the dissimilarity values from 0 to 1, where larger distances correspond to larger dissimilarity values. Second, we constructed a dissimilarity matrix reflecting the AnnaK model based on the mean optimism score of each pair of participants (35). Taking the mean of each pair allowed us to test the hypothesis that only pairs of two optimistic individuals have very similar neural representations when episodic future thinking. These matrix values were normalized to a 0 to 1 scale and then subtracted from 1 to create the dissimilarity matrix. We generated these dissimilarity matrices using the same procedures for both studies. Finally, we used the Mantel test (50) implemented by the vegan package (51) to examine the relationship between each ROI’s neural dissimilarity matrix and model dissimilarity matrices. The Mantel statistic is simply a Spearman’s rank-order correlation between entries of two correlation or dissimilarity matrices. The *P* value is then based on the permutation test. The number of permutations was set to 10,000 in the current study. To account for the multiple comparisons problem that comes with multiple ROIs within the DMN, *P* values were Bonferroni-corrected for the number of ROIs, which was 11.

To investigate potential differences in neural representational patterns between self-referential and partner-referential contexts, we conducted additional analyses in Study 1. We divided the original 8 × 8 neural RDM into two 4 × 4 matrices, one for the self-condition and one for the partner condition. Each 4 × 4 RDM retained only the pairwise dissimilarities relevant to its context. We applied the same procedures as those used in the primary IS-RSAs, including the construction of neural RDMs, the application of optimism-related behavioral models (e.g., the AnnaK and the NN models), and the use of Mantel tests to evaluate correlations. Due to differences in study design, in which Study 2 employed a smaller 6 × 6 RDM with fewer conditions (three conditions per referential target), we did not conduct condition-specific analyses for Study 2.

### INDSCAL.

To investigate the dimensional structure of the neural representations in each ROI, we used INDSCAL to avoid the known distortion of MDS solutions caused by averaging distances across individuals (27, 52). In addition, a major benefit of the INDSCAL model is that it provides solutions that are nonrotatable and, hence, have interpretable dimensions. In addition to defining the future-thinking internal space based on fMRI data, the INDSCAL solution also provides weights for each subject on each dimension of a multidimensional space common to all subjects. The weights indicate the importance of each dimension to each subject. Using this approach, we can investigate whether optimism alters the weights of each dimension in the common spatial representation. This analysis used t-statistic maps to compute pairwise Pearson correlations between neural patterns for all condition pairs. These correlations were then inverted (i.e., 1−Pearson correlation) to create neural RDMs for each study. In Study 1, we constructed an 8 × 8 RDM, whereas in Study 2, a 6 × 6 RDM was used to reflect differences in the number of experimental conditions. For these RDMs, we used the indscal function in the smacof R package (53). This study focused primarily on the MPFC. We have shown the results for other ROIs in *SI Appendix*.

To confirm the robustness of the representational similarity findings that we obtained from both the IS-RSA and INDSCAL analyses, we conducted two supplementary analyses: i) a split-half RSA, which assessed pattern reliability by correlating neural representational patterns between two independent subsets of data created from all possible ways of dividing the 10 runs into halves (5 runs vs. 5 runs, 126 combinations); and ii) a cross-run RSA, which compared neural patterns across independent runs with the aim of controlling for potential run-specific noise and biases. Detailed methodological information and the results of these supplementary analyses are provided in *SI Appendix*, *Supplementary Results*.

## Supplementary Material

Appendix 01 (PDF)

## Data Availability

Behavioral and fMRI data, as well as all analysis scripts used in this study, have been deposited in the Open Science Framework (OSF) and are publicly available at https://osf.io/kgvhj/ (54). All other data are included in the article and/or *SI Appendix*.

## References

[1] S. E. Blackwell , Optimism and mental imagery: A possible cognitive marker to promote well-being? Psychiatry Res. **206**, 56–61 (2013). 23084598 10.1016/j.psychres.2012.09.047 PMC3605581

[2] T. Sharot, A. M. Riccardi, C. M. Raio, E. A. Phelps, Neural mechanisms mediating optimism bias. Nature **450**, 102–105 (2007). 17960136 10.1038/nature06280

[3] L. Brydon, C. Walker, A. J. Wawrzyniak, H. Chart, A. Steptoe, Dispositional optimism and stress-induced changes in immunity and negative mood. Brain Behav. Immun. **23**, 810–816 (2009). 19272441 10.1016/j.bbi.2009.02.018 PMC2715885

[4] M. F. Scheier, C. S. Carver, Optimism, coping, and health: Assessment and implications of generalized outcome expectancies. Health Psychol. **4**, 219–247 (1985). 4029106 10.1037//0278-6133.4.3.219

[5] C. S. Carver, M. F. Scheier, S. C. Segerstrom, Optimism. Clin. Psychol. Rev. **30**, 879–889 (2010). 20170998 10.1016/j.cpr.2010.01.006 PMC4161121

[6] E. C. Baek , In-degree centrality in a social network is linked to coordinated neural activity. Nat. Commun. **13**, 1118 (2022). 35236835 10.1038/s41467-022-28432-3 PMC8891270

[7] I. Brissette, M. F. Scheier, C. S. Carver, The role of optimism in social network development, coping, and psychological adjustment during a life transition. J. Pers. Soc. Psychol. **82**, 102–111 (2002). 11811628 10.1037//0022-3514.82.1.102

[8] R. Iliev, W. M. Bennis, The convergence of positivity: Are happy people all alike? J. Happiness Stud. **24**, 1643–1662 (2023).

[9] E. C. Baek , Lonely individuals process the world in idiosyncratic ways. Psychol. Sci. **34**, 683–695 (2023). 37027033 10.1177/09567976221145316 PMC10404901

[10] P. H. A. Chen, E. Jolly, J. H. Cheong, L. J. Chang, Intersubject representational similarity analysis reveals individual variations in affective experience when watching erotic movies. Neuroimage **216**, 116851 (2020). 32294538 10.1016/j.neuroimage.2020.116851 PMC7955800

[11] D. C. Jangraw , Inter-subject correlation during long narratives reveals widespread neural correlates of reading ability. Neuroimage **282**, 120390 (2023). 37751811 10.1016/j.neuroimage.2023.120390 PMC10783814

[12] E. Komulainen , Escitalopram enhances synchrony of brain responses during emotional narratives in patients with major depressive disorder. Neuroimage **237**, 118110 (2021). 33933596 10.1016/j.neuroimage.2021.118110

[13] D. de Bruin, J. M. van Baar, P. L. Rodríguez, O. FeldmanHall, Shared neural representations and temporal segmentation of political content predict ideological similarity. Sci. Adv. **9**, eabq5920 (2023). 36724226 10.1126/sciadv.abq5920 PMC9891706

[14] K. I. Kim, W. H. Jung, C. W. Woo, H. Kim, Neural signatures of individual variability in context-dependent perception of ambiguous facial expression. Neuroimage **258**, 119355 (2022). 35660000 10.1016/j.neuroimage.2022.119355

[15] J. Sheng , Intersubject similarity in neural representations underlies shared episodic memory content. Proc. Natl. Acad. Sci. U.S.A. **120**, e2308951120 (2023). 37603733 10.1073/pnas.2308951120 PMC10466090

[16] J. M. van Baar, L. J. Chang, A. G. Sanfey, The computational and neural substrates of moral strategies in social decision-making. Nat. Commun. **10**, 1483 (2019). 30940815 10.1038/s41467-019-09161-6 PMC6445121

[17] M. E. Raichle, The brain’s default mode network. Annu. Rev. Neurosci. **38**, 433–447 (2015). 25938726 10.1146/annurev-neuro-071013-014030

[18] R. L. Buckner, D. C. Carroll, Self-projection and the brain. Trends Cogn. Sci. **11**, 49–57 (2007). 17188554 10.1016/j.tics.2006.11.004

[19] D. L. Schacter, D. R. Addis, R. L. Buckner, Remembering the past to imagine the future: The prospective brain. Nat. Rev. Neurosci. **8**, 657–661 (2007). 17700624 10.1038/nrn2213

[20] D. L. Schacter, R. G. Benoit, K. K. Szpunar, Episodic future thinking: Mechanisms and functions. Curr. Opin. Behav. Sci. **17**, 41–50 (2017). 29130061 10.1016/j.cobeha.2017.06.002 PMC5675579

[21] R. N. Spreng, R. A. Mar, A. S. Kim, The common neural basis of autobiographical memory, prospection, navigation, theory of mind, and the default mode: A quantitative meta-analysis. J. Cogn. Neurosci. **21**, 489–510 (2009). 18510452 10.1162/jocn.2008.21029

[22] D. Hassabis , Imagine all the people: How the brain creates and uses personality models to predict behavior. Cereb. Cortex **24**, 1979–1987 (2014). 23463340 10.1093/cercor/bht042 PMC4089378

[23] K. Yanagisawa, E. S. Kashima, Y. Shigemune, R. Nakai, N. Abe, Neural representations of death in the cortical midline structures promote temporal discounting. Cereb. Cortex Commun. **2**, tgab013 (2021). 34296159 10.1093/texcom/tgab013 PMC8152905

[24] X. Wang, W. Men, J. Gao, A. Caramazza, Y. Bi, Two forms of knowledge representations in the human brain. Neuron **107**, 383–393.e5 (2020). 32386524 10.1016/j.neuron.2020.04.010

[25] A. C. Connolly , The representation of biological classes in the human brain. J. Neurosci. **32**, 2608–2618 (2012). 22357845 10.1523/JNEUROSCI.5547-11.2012 PMC3532035

[26] A. Kappes, N. S. Faber, G. Kahane, J. Savulescu, M. J. Crockett, Concern for others leads to vicarious optimism. Psychol. Sci. **29**, 379–389 (2018). 29381448 10.1177/0956797617737129 PMC5858641

[27] Y. Cao, C. Summerfield, H. Park, B. L. Giordano, C. Kayser, Causal inference in the multisensory brain. Neuron **102**, 1076–1087.e8 (2019). 31047778 10.1016/j.neuron.2019.03.043

[28] L. B. Baucom, D. H. Wedell, J. Wang, D. N. Blitzer, S. V. Shinkareva, Decoding the neural representation of affective states. Neuroimage **59**, 718–727 (2012). 21801839 10.1016/j.neuroimage.2011.07.037

[29] B. L. Giordano , The representational dynamics of perceived voice emotions evolve from categories to dimensions. Nat. Hum. Behav. **5**, 1203–1213 (2021). 33707658 10.1038/s41562-021-01073-0 PMC7611700

[30] E. S. Finn , Idiosynchrony: From shared responses to individual differences during naturalistic neuroimaging. Neuroimage **215**, 116828 (2020). 32276065 10.1016/j.neuroimage.2020.116828 PMC7298885

[31] K. K. Assad, M. B. Donnellan, R. D. Conger, Optimism: An enduring resource for romantic relationships. J. Pers. Soc. Psychol. **93**, 285–297 (2007). 17645400 10.1037/0022-3514.93.2.285

[32] S. Srivastava, K. M. McGonigal, J. M. Richards, E. A. Butler, J. J. Gross, Optimism in close relationships: How seeing things in a positive light makes them so. J. Pers. Soc. Psychol. **91**, 143–153 (2006). 16834485 10.1037/0022-3514.91.1.143

[33] R. Böhm, A. Schütz, K. Rentzsch, A. Körner, F. Funke, Are we looking for positivity or similarity in a partner’s outlook on life? Similarity predicts perceptions of social attractiveness and relationship quality. J. Posit. Psychol. **5**, 431–438 (2010).

[34] C. S. Carver, L. A. Kus, M. Scheier, Effects of good versus bad mood and optimistic versus pessimistic outlook on social acceptance versus rejection. J. Soc. Clin. Psychol. **13**, 138–151 (1994).

[35] E. S. Finn, P. R. Corlett, G. Chen, P. A. Bandettini, R. T. Constable, Trait paranoia shapes inter-subject synchrony in brain activity during an ambiguous social narrative. Nat. Commun. **9**, 2043 (2018). 29795116 10.1038/s41467-018-04387-2 PMC5966466

[36] M. Nguyen, T. Vanderwal, U. Hasson, Shared understanding of narratives is correlated with shared neural responses. Neuroimage **184**, 161–170 (2019). 30217543 10.1016/j.neuroimage.2018.09.010 PMC6287615

[37] T. W. Broom, S. Iyer, A. L. Courtney, M. L. Meyer, Loneliness corresponds with neural representations and language use that deviate from shared cultural perceptions. Commun. Psychol. **2**, 40 (2024). 38721125 10.1038/s44271-024-00088-3 PMC11073992

[38] S. Sakamoto, E. Tanaka, A study of the Japanese version of revised life orientation test. Jpn. J. Health Psychol. **15**, 59–63 (2002).

[39] M. F. Scheier, C. S. Carver, M. W. Bridges, Distinguishing optimism from neuroticism (and trait anxiety, self-mastery, and self-esteem): A reevaluation of the life orientation test. J. Pers. Soc. Psychol. **67**, 1063–1078 (1994). 7815302 10.1037//0022-3514.67.6.1063

[40] J. C. Raven, Coloured Progressive Matrices: Sets A, AB, B (Lewis, London, 1962).

[41] J. C. Raven, Guide to Using to the Coloured Progressive Matrices Sets A, AB, B (Lewis, London, 1965).

[42] N. E. Adler, E. S. Epel, G. Castellazzo, J. R. Ickovics, Relationship of subjective and objective social class with psychological functioning: Preliminary data in healthy white women. Health Psychol. **19**, 586–592 (2000). 11129362 10.1037//0278-6133.19.6.586

[43] M. W. Kraus, P. K. Piff, D. Keltner, Social class, sense of control, and social explanation. J. Pers. Soc. Psychol. **97**, 992–1004 (2009). 19968415 10.1037/a0016357

[44] D. A. Feinberg , Multiplexed echo planar imaging for sub-second whole brain FMRI and fast diffusion imaging. PLoS One **5**, e15710 (2010). 21187930 10.1371/journal.pone.0015710 PMC3004955

[45] S. Moeller , Multiband multislice GE-EPI at 7 tesla, with 16-fold acceleration using partial parallel imaging with application to high spatial and temporal whole-brain fMRI. Magn. Reson. Med. **63**, 1144–1153 (2010). 20432285 10.1002/mrm.22361 PMC2906244

[46] J. Xu , Evaluation of slice accelerations using multiband echo planar imaging at 3 T. Neuroimage **83**, 991–1001 (2013). 23899722 10.1016/j.neuroimage.2013.07.055 PMC3815955

[47] M. Misaki, Y. Kim, P. A. Bandettini, N. Kriegeskorte, Comparison of multivariate classifiers and response normalizations for pattern-information fMRI. Neuroimage **53**, 103–118 (2010). 20580933 10.1016/j.neuroimage.2010.05.051 PMC2914143

[48] T. Yarkoni, R. A. Poldrack, T. E. Nichols, D. C. Van Essen, T. D. Wager, Large-scale automated synthesis of human functional neuroimaging data. Nat. Methods **8**, 665–670 (2011). 21706013 10.1038/nmeth.1635 PMC3146590

[49] N. N. Oosterhof, A. C. Connolly, J. V. Haxby, CoSMoMVPA: Multi-modal multivariate pattern analysis of neuroimaging data in matlab/GNU octave. Front. Neuroinformatics **10**, 27 (2016). 10.3389/fninf.2016.00027 PMC4956688 27499741

[50] P. Legendre, L. Legendre, Numerical Ecology (Elsevier, 2012).

[51] J. Oksanen , vegan: Community Ecology Package. R package version 2.6-6.1 (2024). https://CRAN.R-project.org/package=vegan.

[52] F. G. Ashby, W. T. Maddox, W. W. Lee, On the dangers of averaging across subjects when using multidimensional scaling or the similarity-choice model. Psychol. Sci. **5**, 144–151 (1994).

[53] J. de Leeuw, P. Mair, Multidimensional scaling using majorization: Smacof in R. J. Stat. Softw. **31**, 1–30 (2009).

[54] K. Yanagisawa , Data and analysis scripts from “Optimistic people are all alike: Shared neural representations supporting episodic future thinking among optimistic individuals.” Open Science Framework. https://osf.io/kgvhj/. Deposited 29 November 2023. 10.1073/pnas.2511101122 PMC12318172 40690674

