# Supplementary material for: Optimistic people are all alike: Shared neural representations supporting episodic future thinking among optimistic individuals
Source: Proc Natl Acad Sci U S A. 2025 Jul 21;122(30):e2511101122. doi: 10.1073/pnas.2511101122 (PMC12318172; doi:10.1073/pnas.2511101122)
Supplement: Supplementary file 1 — Appendix 01 (PDF) [file pnas.2511101122.sapp.pdf]

PNAS

Supporting Information for
Supplementary online materials for “Optimistic people are all alike: Shared neural representations supporting episodic future thinking among optimistic individuals”

Kuniaki Yanagisawa, Ryusuke Nakai, Kohei Asano, Emiko S. Kashima, Hitomi Sugiura, Nobuhito Abe

corresponding author
Kuniaki Yanagisawa
Email: kuniaki1031@gmail.com

This PDF file includes:

Supporting text
Figures S1 to S16
Tables S1 to S4
SI References

Supporting Information

Supplementary Results

Correlations Between Optimism and Control Variables. Table S2 presents the relationships between optimism and control variables, including age, subjective socioeconomic status (SES) (1, 2), education level, and nonverbal intelligence (assessed using Raven's Coloured Progressive Matrices (3, 4) in Study 2. We found no significant associations between optimism and control variables.

Testing the Anna Karenina (AnnaK) Model While Controlling for the Nearest Neighbor (NN) model and Other Variables. We conducted partial Mantel tests in Studies 1 and 2 to determine whether the AnnaK model explains neural data more effectively than the NN model. In Study 1, significant partial correlations were observed in five regions of interest (ROIs), including the medial prefrontal cortex (MPFC; $\rho = .41$, Bonferroni-corrected $p = .001$), the left parahippocampal gyrus ($\rho = .12$, Bonferroni-corrected $p = .007$), the left middle temporal gyrus ($\rho = .32$, Bonferroni-corrected $p = .025$), the left superior frontal gyrus ($\rho = .29$, Bonferroni-corrected $p = .030$), and the right superior frontal gyrus ($\rho = .32$, Bonferroni-corrected $p = .008$). Similarly, in Study 2, significant correlations were found in five ROIs: the MPFC ($\rho = .38$, Bonferroni-corrected $p = .005$), the precuneus ($\rho = .29$, Bonferroni-corrected $p = .040$), the left middle temporal gyrus ($\rho = .23$, Bonferroni-corrected $p = .044$), the right angular gyrus ($\rho = .28$, Bonferroni-corrected $p = .019$), and the right superior frontal gyrus ($\rho = .31$, Bonferroni-corrected $p = .016$). These findings confirm that the AnnaK model captures representational dissimilarity patterns beyond what the NN model can explain.

We conducted additional partial Mantel tests in Study 2 to assess whether these correlations remained significant after accounting for potential confounding factors. Specifically, we developed separate NN-modeled matrices for each control variable, including age, sex, SES, education, and nonverbal intelligence, using participants' absolute differences in their respective scores. For SES, we also constructed an AnnaK-modeled matrix by calculating the mean SES scores for each participant pair and converting them into a dissimilarity matrix. After incorporating these control matrices into the analyses, the correlations between the true neural dissimilarity matrix and the AnnaK-modeled matrix in the MPFC and the right superior frontal gyrus remained significant (Bonferroni-corrected $ps < .05$). These results underscore the distinct explanatory power of the AnnaK model in accounting for individual differences in neural representations.

Relating Dyad-level Representational Similarity with Optimism. We analyzed representational similarity measures at the dyadic level, following similar methods employed in a prior study (5). This analysis in Study 1 included 37 participants, yielding 666 unique dyads. The analysis in Study 2 involved 50 participants, resulting in 1,225 unique dyads. We computed the Spearman correlation between the neural representational dissimilarity matrix (RDM), in which each entry represented the dissimilarity between a pair of conditions in each ROI for each dyad in both studies. We then applied Fisher z-transformations to the Spearman correlations and normalized the resulting values within each ROI before conducting further analyses.

We analyzed Life Orientation Test-Revised (LOT-R) scores across both studies to examine the relationship between optimism and neural similarity in default mode network (DMN) ROIs. The LOT-R scores ranged from 12 to 25 (median = 19, SD = 3.10) in Study 1. A median split classified 18 participants as highly optimistic and 19 as having low optimism. The LOT-R scores ranged from 7 to 29 (median = 18.5, SD = 4.99) in Study 2, with 25 participants categorized as highly optimistic and 25 as having low optimism. Moreover, the LOT-R scores were approximately normally distributed in both studies. Visually inspecting the histograms (Figure S5) and results of the Shapiro-Wilk test confirmed no significant deviations from normality (Study 1: $W = 0.971$, $p = 0.438$; Study 2: $W = 0.985$, $p = 0.775$).

To relate the dyad-level representational similarity measure with optimism, we transformed the individual-level binarized optimism measure into a dyad-level variable. We labeled dyads as follows: (a) {high, high} if both individuals in a dyad were highly optimistic, (b) {low, low} if both individuals in a dyad had lower optimism, and (c) {high, low} if one individual in a dyad was highly optimistic while the other was less optimistic. To relate this dyad-level optimism measure to representational similarity, we employed the methodology from prior studies (5, 6) and fitted linear mixed-effects models with crossed random effects using lme4 and lmerTest R packages (7, 8). For additional details, encompassing information regarding the approach, see Baek et al. (5) and Chen et al. (6). Before conducting statistical inference, we manually adjusted the degrees of freedom to N - k, where N represents the number of unique observations, and k is the number of fixed effects in the model. All findings that we report in the present article use the corrected number of degrees of freedom. For each ROI, we first fit a mixed-effects model, with representational similarities in the corresponding brain region as the dependent variable, the dyad-level binarized optimism variable as the independent variable, and random intercepts for each individual in a dyad. We then conducted planned contrasts using the emmeans R package (9) to identify brain regions in which the inter-subject correlation (ISC) of neural representations is increased in pairs of optimistic individuals: ISC{high, high} > ISC{low, low}, ISC{high, high} > ISC{high, low}, and ISC{high, low} > ISC{low, low}. We z-scored all variables to yield standardized coefficients (β) as outputs. We applied a Bonferroni correction to p-values for multiple comparisons, with a significance level set at $p < .05$.

We found that dyads in which both individuals were highly optimistic had greater ISCs than dyads in which both members were less optimistic (Table S3) in the following brain regions in Study 1: MPFC, left parahippocampal gyrus, left middle temporal gyrus, and right superior frontal gyrus. The ISCs in these regions were also greater in {high, high} dyads than in {high, low} dyads. Study 2 showed a similar pattern, with {high, high} dyads exhibiting significantly greater ISCs in overlapping regions, including the MPFC and right superior frontal gyrus (Table S4). As in Study 1, the ISCs in these regions were also greater in {high, high} dyads than in {high, low} dyads.

Contrast-Specific IS-RSA Results. As an exploratory investigation, we conducted additional intersubject representational similarity analysis (IS-RSA) focusing specifically on the MPFC to determine whether the observed neural representational dissimilarity associated with optimism scores was driven by specific condition contrasts, such as positive vs. neutral and negative vs. neutral. We extracted pairwise dissimilarity values from individual participants' neural RDMs to examine these contrasts. We conducted separate analyses for the self-condition and partner condition to investigate whether the effects differed depending on the referential target. After extracting the contrast-specific dissimilarity values, we sorted them based on participants' optimism scores to assess whether neural dissimilarity patterns aligned with optimism levels. We computed Euclidean distance matrices for each contrast to represent the neural dissimilarity structure across participants. We applied optimism-related behavioral models, including the AnnaK and NN models, to evaluate these relationships.

We performed Mantel tests for each contrast-specific distance matrix to determine whether neural dissimilarity aligned with these behavioral models. The Mantel statistic (Spearman's correlation) quantified the relationship between the neural distance matrices and the AnnaK model. In Study 1, the IS-RSA analyses for the positive vs. neutral and negative vs. neutral contrasts did not yield significant correlations with the AnnaK or NN models. In Study 2, we conducted similar analyses. Although we did not find significant results for the partner condition in either contrast, the positive vs. neutral contrast in the self-condition showed alignment with the AnnaK model ($\rho = .14$, $p = .013$). The negative vs. neutral contrast did not yield significant results. The findings from contrast-specific analyses were less consistent and robust than the main IS-RSA results, which captured broader representational patterns across all conditions. These results highlight the importance of focusing on comprehensive analyses rather than isolated condition contrasts to better understand the neural correlates of optimism.

Modified AnnaK Model. The original AnnaK model was constructed by calculating the mean optimism score of each participant pair and transforming it into a similarity matrix, in which higher values represented increased similarity. However, this approach assumes that the mean fully captures the relationship between two optimism scores, potentially overlooking differences within each pair. We conducted a supplementary analysis using an alternative model that explicitly incorporates differences between scores to address the concern that averaging optimism scores might fail to account for individual variability. This revised model penalizes pairs with large optimism score discrepancies, adjusting the similarity values accordingly. Specifically, we calculated the similarity between the two participants using a weighted combination of their mean optimism score and the absolute difference between their scores. A penalty parameter (α = 0.5) controlled the extent to which differences reduced the similarity value. The resulting similarity matrix was normalized to ensure all values ranged between 0 and 1. For example, in a pair where one participant had an optimism score of 25 and the other had a score of 15, the mean optimism score would be 20. However, the absolute difference of 10 suggests lower similarity. By applying the penalty parameter, we adjusted the similarity value downward, ensuring that pairs with large optimism discrepancies are not misrepresented as similar based solely on their mean score. This approach provides a more accurate representation of pairwise similarity by integrating the mean and the variability between scores (Figure S6).

In Study 1, the alternative model incorporating optimism score differences yielded a significant correlation between the true neural dissimilarity matrix and the adjusted AnnaK-modeled matrix in four ROIs: the MPFC (ρ = .42, Bonferroni-corrected p = .002), left parahippocampal gyrus (ρ = .17, Bonferroni-corrected p = .002), left middle temporal gyrus (ρ = .35, Bonferroni-corrected p = .012), and right superior frontal gyrus (ρ = .27, Bonferroni-corrected p = .046), whereas the other ROIs did not show significant correlations (Bonferroni-corrected ps > .050). In Study 2, the alternative model similarly revealed significant correlations in the MPFC (ρ = .41, Bonferroni-corrected p = .001), left parahippocampal gyrus (ρ = .11, Bonferroni-corrected p = .013), precuneus (ρ = .31, Bonferroni-corrected p = .016), and right superior frontal gyrus (ρ = .31, Bonferroni-corrected p = .014), whereas the other ROIs indicated no significant correlations (Bonferroni-corrected ps > .050). These findings suggest that the alternative model, which incorporates differences within each pair, effectively highlights that pairs with higher combined optimism scores tend to exhibit more neural similarity than pairs in which one or both individuals have lower optimism.

Lower-Dimensional Representation of Neural Activities in the Other ROIs. We conducted individual differences multidimensional scaling (INDSCAL) analyses for additional ROIs, including the cerebellum, angular gyrus, parahippocampal gyrus, middle temporal gyrus, precuneus, and superior frontal gyrus. Neural RDMs were extracted and analyzed separately for each ROI, using 8 × 8 matrices in Study 1 and 6 × 6 matrices in Study 2. To simplify data presentation, we visualized and described only the first two INDSCAL dimensions in the supplementary figures (Figures S7 and S8).

In Study 1, these two dimensions were derived from three-dimensional solutions, whereas in Study 2, we selected two-dimensional solutions to capture the core neural representational structure. We performed multidimensional scaling (MDS) on the pairwise Mantel test results to evaluate further the similarity between the representational structures of the MPFC and other ROIs. We computed a dissimilarity matrix for each ROI based on Euclidean distances between conditions in the two-dimensional common space derived from INDSCAL. We then used Mantel tests to compare the MPFC dissimilarity matrix with those of the other ROIs. We applied Bonferroni correction to correct for multiple comparisons, setting the number of comparisons to the total number of ROIs minus one (i.e., excluding the MPFC). The resulting Mantel correlation coefficients were visualized in a two-dimensional MDS plot, highlighting patterns of ROIs with similar representational structures (Figure S9).

In Study 1, specific ROIs, such as the left angular gyrus and the left superior frontal gyrus, exhibited representational structures similar to those of the MPFC. Specifically, the first INDSCAL dimension reflected emotional valence, whereas the second dimension corresponded to the referential target (self vs. partner). Mantel tests confirmed significant correlations between the MPFC and several ROIs, including the left angular gyrus, right angular gyrus, right middle temporal gyrus, right superior frontal gyrus, and left superior frontal gyrus (Bonferroni-corrected ps $< .050$). In Study 2, the two-dimensional INDSCAL spaces for the ROIs exhibited variability in their representational structures. The right middle temporal gyrus and right superior frontal gyrus showed dimensions that reflected valence and referential target, similar to the MPFC. However, other ROIs exhibited less interpretable representational patterns. Mantel tests, adjusted for multiple comparisons, confirmed significant correlations between the MPFC, right middle temporal gyrus, and right superior frontal gyrus (Bonferroni-corrected ps $< .050$). These findings suggest that while some ROIs share a representational structure with the MPFC, particularly along dimensions reflecting valence and referential target, other brain regions may encode additional aspects of episodic future thinking.

Univariate Analysis of MPFC Activity. To determine whether the observed IS-RSA and INDSCAL results in the MPFC could be attributed to univariate effects, we conducted additional analyses using percentage signal changes (i.e., univariate activation levels) for each experimental condition. We extracted percentage signal changes from the MPFC ROI using MarsBaR software (10).

In Study 1, we conducted a 2 × 4 repeated-measures analysis of variance (ANOVA) to examine the effects of referential target (self vs. partner) and emotion (death, negative, neutral, positive). The analysis revealed significant main effects for both referential targets ($p < .001$) and emotion ($p < .001$), whereas the interaction effect was not significant ($p = .155$). Figure S10a illustrates the distribution of MPFC activity across conditions. Regarding the main effect of emotion, the death condition elicited significantly less activation than the negative (Bonferroni-corrected $p = .012$), neutral (Bonferroni-corrected $p < .001$), and positive conditions (Bonferroni-corrected $p = .033$). Additionally, the neutral condition showed significantly more activation than the positive condition (Bonferroni-corrected $p = .035$). For the main effect of the referential target, self-related conditions produced significantly less activation than partner-related conditions ($p < .001$).
In Study 2, we conducted a 2 × 3 repeated-measures ANOVA to assess the effects of referential target (self vs. partner) and emotion (negative, neutral, positive). The analysis revealed significant main effects for both referential target ($p = .016$) and emotion ($p < .001$), as well as a significant interaction effect between referential target and emotion ($p < .001$). Figure S10b visualizes the distribution of MPFC activity across conditions. The main effect of emotion indicated that both positive and negative conditions produced significantly higher activation than the neutral condition (Bonferroni-corrected ps $< .001$). For the main effect of the referential target, self-related conditions elicited significantly less activation than partner-related conditions ($p = .016$). For the interaction effect, in the self-condition, both the positive and negative conditions exhibited significantly higher activation than the neutral condition (Bonferroni-corrected ps $< .001$). However, we observed no significant difference in the partner condition (Bonferroni-corrected ps $> .050$).

Additionally, we conducted a univariate analysis to assess whether optimism levels moderated the contrasts of positive vs. neutral and negative vs. neutral in Studies 1 and 2. The results indicated that the MPFC activity in the partner condition for the negative vs. neutral contrast was significantly higher in individuals with lower optimism levels ($p = .046$) only in Study 1. However, neither study found any moderation effects of optimism for other contrasts or conditions (ps $> .050$). We performed similar analyses for other ROIs but detected no significant effects of optimism for the positive vs. neutral or negative vs. neutral contrasts in any of these brain regions (ps $> .050$).

Univariate Effects on IS-RSA and INDSCAL. We computed Euclidean distance matrices using the univariate data to represent each participant's pairwise dissimilarities between conditions. These Euclidean distances captured differences in activity levels between conditions, providing a straightforward measure of dissimilarity based on univariate signal changes. We then subjected these distance matrices to IS-RSA and examined their association with theoretical models of representational similarity, including the AnnaK and the NN models.

We used Mantel tests (Spearman method, 10,000 permutations) for each ROI to compare the pairwise Euclidean distance matrices with the AnnaK and NN models. We applied a Bonferroni correction to adjust p-values to correct for multiple comparisons across the 11 ROIs. No significant correlations emerged in Study 1 between the univariate-based distance matrices and the AnnaK or NN models in any ROI (Bonferroni-corrected ps > .050). In Study 2, the AnnaK model again did not show significant correlations (Bonferroni-corrected ps > .050). However, the NN model yielded significant correlations in three ROIs: the precuneus (ρ = .19, Bonferroni-corrected p = .031), right middle temporal gyrus (ρ = .20, Bonferroni-corrected p = .047), and left middle temporal gyrus (ρ = .19, Bonferroni-corrected p = .038).

We also compared the univariate-based distance matrices with MVPA-based distance matrices, which we constructed following the procedure outlined in Figure 4. Using Mantel tests (Spearman method, 10,000 permutations) and applying Bonferroni correction for multiple comparisons across the 11 ROIs, we found low correlations between univariate-based and MVPA-based distance matrices in Studies 1 and 2 (ρ range: -0.079 to 0.145). We observed no significant effects after correction in any ROI (Bonferroni-corrected p > .050). These findings suggest that univariate activation levels alone cannot account for the representational patterns observed in the MVPA-based IS-RSA analyses.

Additionally, we subjected the univariate-based distance matrices to INDSCAL to explore the lower-dimensional representation of neural activities in 11 ROIs across Studies 1 and 2. We determined that a three-dimensional INDSCAL model was the optimal representation for Study 1, whereas we selected a two-dimensional model for Study 2. However, as illustrated in Figures S11 and S12, the univariate-based INDSCAL analyses failed to capture the valence (positive vs. negative) or referential target (self vs. partner) distinctions prominently observed in the MVPA-based INDSCAL analyses.

The Relationship between INDSCAL Weights and Participant Similarity in the MPFC. To clarify the relationship between the INDSCAL dimensional weights (particularly Dimension 1, reflecting valence) and the intersubject similarity structure derived from the MPFC neural representational patterns, we conducted an additional visualization and correlation analysis. Specifically, participants' weights on Dimension 1 were visualized in relation to their spatial positions within the participant-similarity MDS space. As illustrated in Figure S13, participants who were characterized by higher Dimension 1 weights (indicated by warmer colors) consistently clustered near the region that was identified as exhibiting the highest density (marked by a cross), thus suggesting that participants who exhibited closer alignment with the common valence dimension were also characterized by shared MPFC neural representations. In contrast, participants who were characterized by lower Dimension 1 weights (cooler colors) exhibited greater dispersion, reflecting weaker alignment with the common dimension and more idiosyncratic MPFC neural representations. Statistical analysis confirmed this visual observation, particularly by revealing robust negative correlations between participants' Dimension 1 weights and their distance from the highest-density region identified by Gaussian kernel density estimation in both Study 1 (ρ = -.87, p < .001) and Study 2 (ρ = -.46, p < .001). These findings confirm that higher INDSCAL weights, which are associated with optimistic individuals, reflect greater neural representational convergence in the MPFC.

Split-Half RSA. To address concerns regarding the interpretability of the correlation distance measure used in our primary analyses (e.g., 11), we conducted a supplementary split-half RSA

implemented via CoSMoMVPA (12). This analysis explicitly tested whether the neural activity patterns associated with each experimental condition were stable and reproducible within individual participants, thereby confirming that the observed representational similarities reflect meaningful neural signals (rather than noise or unreliable fluctuations). Specifically, following previous studies (e.g., 13), we divided the dataset into two independent subsets in all possible ways (5 runs vs. 5 runs, 126 combinations), and computed the correlation matrices as described below for each split before averaging them. For each participant and ROI, we extracted multivariate fMRI activity patterns separately from these two subsets for each experimental condition. We then computed Pearson correlation coefficients between corresponding activity patterns (i.e., the same conditions between the subsets), thereby producing a condition-by-condition correlation matrix for each participant. These correlation values were subsequently normalized via Fisher's Z-transformation. To quantify the overall stability of the neural activity patterns, we calculated a weighted contrast value for each participant. This metric reflected the representational structure that we expected to observe: correlations between identical conditions across subsets should be high, whereas correlations between different conditions should be low. Specifically, we created a contrast matrix in which diagonal elements (i.e., correlations between identical conditions) were assigned positive weights, whereas off-diagonal elements (i.e., correlations between different conditions) were assigned negative weights. The multiplication of each Fisher's Z-transformed correlation matrix by this contrast matrix yielded a weighted matrix, and the sum of all the elements included in this weighted matrix provided the weighted contrast value for each participant. Importantly, higher weighted contrast values indicate higher levels of reproducibility and reliability with respect to the neural activity patterns across independent subsets of data, thus providing strong evidence for meaningful neural representations. In contrast, values near zero indicate unstable, unreliable neural patterns that are likely dominated by noise.

In both Studies 1 and 2, no significant differences in weighted contrast values between high and low optimism groups were observed in any ROIs (Bonferroni-corrected $ps > .702$). Critically, to address concerns regarding noise-driven correlations directly, we subsequently examined pattern stability specifically within participants in the low-optimism group. If the neural patterns of participants who exhibited low levels of optimism were primarily driven by noise, the corresponding weighted contrast values would be near zero or negative, thus indicating instability. Conversely, significantly positive weighted contrast values would indicate that these participants exhibit stable, meaningful neural patterns. In Study 1, we performed one-sample t tests (one-tailed) among participants in the low-optimism group to determine whether the weighted contrast values were significantly greater than zero. The results revealed significant pattern stability across multiple ROIs (left superior frontal gyrus: $t = 6.61$, Bonferroni-corrected $p < .001$; left angular gyrus: $t = 5.61$, Bonferroni-corrected $p < .001$; left middle temporal gyrus: $t = 5.18$, Bonferroni-corrected $p < .001$; precuneus: $t = 6.04$, Bonferroni-corrected $p < .001$; right superior frontal gyrus: $t = 4.31$, Bonferroni-corrected $p = .002$; right angular gyrus: $t = 4.17$, Bonferroni-corrected $p = .003$; right middle temporal gyrus: $t = 3.04$, Bonferroni-corrected $p = .039$; MPFC: $t = 4.65$, Bonferroni-corrected $p = .001$). In Study 2, the pattern stability observed among low-optimism participants was even stronger, and significant stability was observed in all ROIs with the exception of the bilateral parahippocampal gyrus (cerebellum: $t = 4.60$, Bonferroni-corrected $p < .001$; left superior frontal gyrus: $t = 9.64$, Bonferroni-corrected $p < .001$; left angular gyrus: $t = 6.88$, Bonferroni-corrected $p < .001$; left middle temporal gyrus: $t = 5.64$, Bonferroni-corrected $p < .001$; precuneus: $t = 6.89$, Bonferroni-corrected $p < .001$; right superior frontal gyrus: $t = 7.69$, Bonferroni-corrected $p < .001$; right angular gyrus: $t = 6.73$, Bonferroni-corrected $p < .001$; right middle temporal gyrus: $t = 7.47$, Bonferroni-corrected $p < .001$; MPFC: $t = 7.63$, Bonferroni-corrected $p < .001$). These results clearly indicate that neural representations in individuals who exhibit low levels of optimism are highly reproducible and meaningful (rather than reflecting noisy or unstable patterns).

Cross-Run RSA. To validate the robustness of our representational similarity findings in further detail, we conducted an additional, complementary cross-run RSA. While the split-half RSA

described above demonstrates the reproducibility of activity patterns within specific experimental conditions, cross-run RSA specifically evaluates the consistency and generalizability of abstract neural representations across independent subsets of data (14). Following the same approach described in the split-half RSA, we considered all possible ways of dividing the 10 runs into two independent subsets (5 runs vs. 5 runs; 126 combinations). For each participant and ROI, we computed a cross-run correlation-based RDM. Specifically, for each pair of experimental conditions and for each of the 126 split-half combinations, we calculated correlation distances (1 minus the Pearson correlation coefficient) between the activity patterns pertaining to condition *i* in one subset of five runs and condition *j* in the complementary subset of five runs, as well as the reverse pairing (condition *j* in the first subset and condition *i* in the second subset). We averaged these two distance values to create a symmetric, cross-run RDM for each split-half combination. We then averaged the resulting 126 RDMs, producing a single robust cross-run RDM per participant. We explicitly set the diagonal elements of this RDM to zero because the dissimilarity between identical conditions should inherently be zero. This cross-validated approach helped us mitigate the potential influence of condition-irrelevant noise and biases, thereby enhancing the interpretability and robustness of our primary findings. However, cross-run RSA inherently restricts comparisons of representational similarity to pairs of conditions that are presented across separate halves of the data, thus preventing within-subset comparisons and limiting the ability to obtain a full overview of the fine-grained representational geometry among all conditions to some degrees. Furthermore, episodic future thinking paradigms—such as the approach used here—often present each unique scenario only once throughout the entire experiment (e.g., 15). Under these conditions, conventional RSA is more practically suited to the task of capturing subtle, nuanced representational structures, particularly those that are sensitive to unique stimulus content or single-trial variability. Therefore, given that our primary aim was to characterize representational differences both within and between emotional conditions precisely, we primarily employed conventional (i.e., noncross-validated) RSA in the main analyses. Nevertheless, cross-run RSA remains a valuable approach to the task of assessing generalizable neural representations across independent subsets of data.

We then reexamined the associations between optimism and neural representational similarity via the cross-run RSA approach. We employed the same procedure that we used in our primary analyses. Accordingly, first, we constructed participant-by-participant neural dissimilarity matrices for each DMN ROI, in which context we ordered participants by reference to their optimism scores (see Figure S14a and S14c for the MPFC). We employed MDS on the basis of these cross-run matrices, once again revealing that more optimistic participants clustered closely together, whereas less optimistic participants appeared to be more dispersed (see Figure S14b and S14d). In line with our original results, Spearman correlation analyses revealed significant negative correlations between participants' optimism scores and their distance from the densest region identified within the MDS space in both studies (Study 1: $\rho = -.68$, $p < .001$; Study 2: $\rho = -.29$, $p = .042$).

Furthermore, we conducted the IS-RSA on the basis of the NN and AnnaK models by reference to the cross-run RSA matrices. Once again, no significant correlations were observed with regard to the NN model in any of the ROIs (all Bonferroni-corrected ps > .05). However, in line with our primary analyses, significant correlations between the AnnaK model and the cross-run neural dissimilarity matrices were observed in the MPFC (Study 1: $\rho = .47$, Bonferroni-corrected $p = .001$), precuneus ($\rho = .37$, Bonferroni-corrected $p = .009$), left angular gyrus ($\rho = .33$, Bonferroni-corrected $p = .029$), left middle temporal gyrus ($\rho = .33$, Bonferroni-corrected $p = .018$), left superior frontal gyrus ($\rho = .32$, Bonferroni-corrected $p = .014$), and right superior frontal gyrus ($\rho = .30$, Bonferroni-corrected $p = .014$). In Study 2, significant correlations were observed in the MPFC ($\rho = .34$, Bonferroni-corrected $p = .016$), left middle temporal gyrus ($\rho = .24$, Bonferroni-corrected $p = .021$), and the right superior frontal gyrus ($\rho = .28$, Bonferroni-corrected $p = .037$). These results provide additional support for the robustness of the observed relationship between optimism and neural representational similarity, thus indicating that

optimistic individuals consistently exhibit shared neural representations with one another, even when representational similarity is assessed by reference to independent subsets of data.

We also repeated the INDSCAL analysis by reference to neural RDMs that were obtained via the cross-run RSA approach. Similar to our primary analyses, we examined the lower-dimensional structure of neural representations in the MPFC (Figure S15a and S15c). In Study 1, we tested INDSCAL solutions with 2, 3, and 4 dimensions, which resulted in stress values of .25, .15, and .11, respectively. The three-dimensional model exhibited good interpretability and fit, in line with the original analysis. The first dimension once again corresponded strongly to valence (ρ = -.83, p = .008, one-tailed), whereas the second dimension was related to the referential target (ρ = -.76, p = .014, one-tailed). In Study 2, the three-dimensional model (stress = .10) established an optimal balance between interpretability and fit. Dimension 1 reflected emotional valence (ρ = -.71, p = .068, one-tailed), although this correlation was marginally significant, and Dimension 2 once again robustly differentiated between targets that referred to the self and those that referred to the partner (ρ = -.87, p = .011, one-tailed). The participant-specific weights obtained via the cross-run INDSCAL analyses also exhibited similar patterns to those reported in our primary results (Figure S15b and S15d). The optimism scores were significantly correlated with the Dimension 1 weights in Study 1 (ρ = .68, $p < .001$), and a similar pattern was observed in Study 2 (ρ = .38, p = .006). These findings indicate that the abstract dimensional structure underlying optimistic individuals' neural representations—which is characterized by a higher level of differentiation between positive and negative events—remains robust and generalizable across independent subsets of data.

Behavioral Data. Following stimulus selection, another behavioral experiment was conducted to further assess the stimulus set and validate the measures. A group of 20 married individuals (10 males and 10 females; age range: 30–45 years, M = 40.30 years), who did not participate in the fMRI study, participated in the behavioral task on two different days (mean interval = 8.1 days, range: 1–18 days). On Day 1, they were explicitly instructed to project themselves into each described future event (positive, neutral, negative, or death-related) and vividly imagine it as something that would genuinely occur in the future, experiencing the situation mentally. On Day 2, participants were instructed to project their partner into the same events as those from Day 1 and vividly imagine these events occurring to their partner in the future. In both tasks, they rated each event on an 8-point Likert scale in terms of (1) emotional valence (1 = “extremely positive” to 8 = “extremely negative”), (2) arousal (1 = “not arousing” to 8 = “very arousing”), (3) semantic death relevance (1 = “not related at all” to 8 = “very strongly related”), (4) vividness (i.e., “To what extent did you imagine the event clearly?”, 1 = “not at all” to 8 = “very clearly”), (5) projection (i.e., “To what extent were you able to project yourself (or your partner) into the episode as if you were actually there?”, 1 = “not at all” and 8 = “very strongly”), (6) subjective temporal distance (i.e., “How far is this event from you in terms of time?”, 1 = “very near future” and 8 = “very distant future”), and (7) self-relevance (i.e., “To what extent do you feel that this event is relevant to you?”, 1 = “not at all” and 8 = “very strongly”). These scores were subjected to repeated-measures analyses of variance (ANOVAs) with the referential target (self or partner) and emotional valence (positive, neutral, negative, or death-related) as within-subjects variables. All multiple comparisons were corrected with the Bonferroni correction.

Repeated-measures ANOVAs were conducted for each dependent variable, with emotional valence (positive, neutral, negative or death-related) and referential target (self or partner) as independent variables (Figure S16). For the valence scores, there was a main effect of the emotional valence, $F(3, 57) = 841.03$, $p < .001$, $\eta 2G = .957$. The positive events were more positive in valence than the neutral, $t = 14.14$, $p < .001$, negative, $t = 40.79$, $p < .001$, and death-relevant events, $t = 41.51$, $p < .001$. The emotional valence scores of the negative events and death-related events were comparable, $t = 0.72$, $p = 1.000$. We did not find a significant main effect of referential target, $F(1, 19) = 1.62$, $p = .218$, $\eta 2G = .005$. However, we did observe a significant referential target × emotional valence interaction, $F(3, 57) = 21.44$, $p < .001$, $\eta 2G$

= .180. Specifically, the valence scores in the self- and partner-referential conditions significantly differed for positive, $t = 6.01$, $p < .001$, and neutral events, $t = 5.30$, $p < .001$.

For the arousal score, there was a main effect of emotional valence, $F(3, 57) = 21.01$, $p < .001$, $\eta 2G = .357$. The arousal levels of positive, negative, and death-related events were similar, ps > 0.950; these arousal levels were all greater than those of neutral events ($t = 7.22$, $p < .001$; $t = 5.80$, $p < .001$; and $t = 6.08$, $p < .001$, respectively). We found an additional main effect of referential target, $F(1, 19) = 6.09$, $p < .05$, $\eta 2G = .013$, reflecting greater arousal of participants in response to future events involving themselves rather than their partners. We also found a significant referential target × emotional valence interaction, $F(3, 57) = 16.20$, $p < .001$, $\eta 2G = .035$. There were significant differences between arousal scores in the self- and partner-referential conditions for positive, $t = 6.14$, $p < .001$, and neutral events, $t = 2.14$, $p < .05$. In addition, while the pattern of the results for the partner-referential condition was similar to the main effect of affective information, the arousal scores of positive events were greater than those of negative events, $t = 2.78$, $p < .05$, and those of death-related events in the self-referential condition, $t = 2.97$, $p < .05$.

For the death relevance score, there was only a main effect of emotional valence, $F(3, 57) = 365.33$, $p < .001$, $\eta 2G = .900$; there was no significant main effect of referential target nor was there a significant referential target × emotional valence interaction, $F(1, 19) = 4.23$, $p = .054$, $\eta 2G = .008$, and $F(3, 57) = 1.05$, $p = .358$, $\eta 2G = .005$, respectively. The death relevance scores of death-related events were greater than those of negative, $t = 20.88$, $p < .001$, neutral, $t = 28.67$, $p < .001$, and positive events, $t = 28.54$, $p < .001$. Furthermore, the death relevance scores of negative events were greater than those of neutral events, $t = 7.79$, $p < .001$, and positive events, $t = 7.66$, $p < .001$.

For the vividness score, there was only a significant main effect of emotional valence, $F(3, 57) = 32.59$, $p < .001$, $\eta 2G = .291$; the main effect of referential target and the referential target × emotional valence interaction were not significant, $F(1, 19) = 4.02$, $p = .060$, $\eta 2G = .020$, and $F(3, 57) = 2.55$, $p = .099$, $\eta 2G = .010$, respectively. The vividness scores of positive and neutral events were comparable, $t = 2.67$, $p = .059$, and both were greater (i.e., more vivid) than those of negative events ($t = 5.16$, $p < .001$ and $t = 7.83$, $p < .001$, respectively) and death-related events ($t = 5.62$, $p < .001$ and $t = 8.29$, $p < .001$). The vividness scores of negative and death-related events were comparable, $t = 0.46$, $p = 1.000$.

For the projection score, there was a significant main effect of emotional valence, $F(3, 57) = 27.06$, $p < .001$, $\eta 2G = .256$. The projection scores of positive and neutral events were comparable, $t = 1.95$, $p = .340$, with both scores being greater than those of negative events ($t = 5.23$, $p < .001$ and $t = 7.18$, $p < .001$, respectively) and death-related events ($t = 5.26$, $p < .001$ and $t = 7.21$, $p < .001$). We found a significant main effect of referential target, $F(1, 19) = 4.79$, $p < .05$, $\eta 2G = .025$, reflecting participants' overall greater projection of themselves into their own future events than those of their partner. We did not find a significant referential target × emotional valence interaction, $F(3, 57) = 2.44$, $p = .112$, $\eta 2G = .012$.

For the subjective temporal distance, there was a significant main effect of emotional valence, $F(3, 57) = 110.69$, $p < .001$, $\eta 2G = .670$. The temporal distances of negative and death-related events were comparable, $t = 0.65$, $p = 1.000$, but both were greater than those of neutral events ($t = 14.68$, $p < .001$ and $t = 15.32$, $p < .001$, respectively) and positive events ($t = 9.27$, $p < .001$ and $t = 9.92$, $p < .001$). Neutral events had lower temporal distances than positive events, $t = 5.41$, $p < .001$. We also found a significant main effect of referential target, $F(1, 19) = 7.05$, $p < .05$, $\eta 2G = .035$; that is, participants imagined their own future events as closer in temporal proximity than those of their partner. We did not find a significant referential target × emotional valence interaction, $F(3, 57) = 0.56$, $p = .560$, $\eta 2G = .003$. We note that negative events ($M = 6.46$) and death-related events ($M = 6.59$) were perceived as occurring farther in the future, neutral events ($M = 3.34$) as occurring closer in time, and positive events ($M = 4.49$) as moderately distant.

For the self-relevance score, there was a significant main effect of emotional valence, $F(3, 57) = 7.88$, $p < .001$, $\eta 2G = .076$. The self-relevance scores of positive, neutral and death-related events were comparable, ts < 1.65, ps > .63 and each greater than those of negative events ($t = 3.49$, $p < .01$; $t = 4.66$, $p < .001$; and $t = 3.01$, $p < .05$, respectively). We also found a significant main effect of referential target, $F(1, 19) = 5.85$, $p < .05$, $\eta 2G = .067$, reflecting overall greater self-relevance of the partner's future events. The referential target × emotional valence interaction was significant, $F(3, 57) = 45.31$, $p < .001$, $\eta 2G = .353$. Both negative and death-related events that impacted oneself had lower self-relevance scores than those that impacted one's partner ($t = 5.58$, $p < .001$ and $t = 6.87$, $p < .001$, respectively). In contrast, neutral events that impacted oneself had higher self-relevance scores than those that impacted one's partner, $t = 4.77$, $p < .001$. In addition, the self-relevance scores of neutral events were greater than those of positive events, $t = 3.31$, $p < .01$, negative events, $t = 9.76$, $p < .001$, and death-related events, $t = 8.54$, $p < .001$, in the self-referential condition. The self-relevance scores of positive events were greater than those of negative events, $t = 6.45$, $p < .001$, and death-related events, $t = 5.22$, $p < .001$. In contrast, the self-relevance scores of death-related events were greater than those of positive events, $t = 4.58$, $p < .001$, neutral events, $t = 6.29$, $p < .001$, and negative events, $t = 2.88$, $p < .05$, in the partner referential condition. Furthermore, the self-relevance scores of negative events were greater than those of neutral events in the partner-referential condition, $t = 3.42$, $p < .01$.

SI References

1. N. E. Adler, E. S. Epel, G. Castellazzo, J. R. Ickovics, Relationship of subjective and objective social class with psychological functioning: Preliminary data in healthy White women. *Health Psychol. 19, 586–592 (2000)*.
2. M. W. Kraus, P. K. Piff, D. Keltner, Social class, sense of control, and social explanation. J*. Pers. Soc. Psychol. 97(6), 992–1004 (2009)*.
3. J. C. Raven, Coloured Progressive Matrices: Sets A, AB, B. Lewis, London (1962).
4. J. C. Raven, Guide to using to the Coloured Progressive Matrices Sets A, AB, B. Lewis, London (1965).
5. E. C. Baek et al., Lonely individuals process the world in idiosyncratic ways. *Psychol. Sci. 34, 683–695 (2023).*
6. G. Chen, P. A. Taylor, Y. W. Shin, R. C. Reynolds, R. W. Cox, Untangling the relatedness among correlations, Part II: Inter-subject correlation group analysis through linear mixed-effects modeling. *Neuroimage*. 147, 825–840 (2017).
7. D. Bates, M. Mächler, B. Bolker, S. Walker, Fitting linear mixed-effects models using lme4*. J. Stat. Softw*. 67, 1–48 (2015).
8. A. Kuznetsova, P. B. Brockhoff, R. H. B. Christensen, lmertest package: Tests in linear mixed effects models*. J. Stat. Softw*. 82, 1–26 (2017).
9. R. V. Lenth, emmeans: Estimated Marginal Means, aka Least-Squares Means. R package version 1.10.5 (2024). https://CRAN.R-project.org/package=emmeans.
10. M. Brett et al., Region of interest analysis using an SPM toolbox [abstract] Presented at the 8th International Conference on Functional Mapping of the Human Brain, June 2–6, Sendai, Japan. Available on CD-ROM in NeuroImage. Vol 16, No 2 (2002).
11. A. Walther et al., Reliability of dissimilarity measures for multi-voxel pattern analysis. *Neuroimage*, 137, 188-200 (2016).
12. N. N. Oosterhof, A. C. Connolly, J. V. Haxby, CoSMoMVPA: Multi-modal multivariate pattern analysis of neuroimaging data in matlab/GNU octave. *Front. Neuroinformatics*. 10, 27 (2016).
13. S. H. Lee, D. J. Kravitz, C. I. Baker, Disentangling visual imagery and perception of real-world objects. *Neuroimage*. 59, 4064–4073 (2012).
14. A. B. Baram, T. H. Muller, H. Nili, M. M. Garvert, T. E. J. Behrens, Entorhinal and ventromedial prefrontal cortices abstract and generalize the structure of reinforcement learning problems. *Neuron*, 109(4), 713-723 (2021).
15. T. Sharot, A. M. Riccardi, C. M. Raio, E. A. Phelps, Neural mechanisms mediating optimism bias. *Nature*. 450, 102–105 (2007).
16. M. Allen et al., Raincloud plots: A multi-platform tool for robust data visualization. *Wellcome Open Res.* 4, 63 (2021).
17. K. Yanagisawa, E. S. Kashima, Y. Shigemune, R. Nakai, N. Abe, Neural representations of death in the cortical midline structures promote temporal discounting. Cereb. Cortex Commun. 2, tgab013 (2021).
18. T. Yarkoni, R. A. Poldrack, T. E. Nichols, D. C. Van Essen, T. D. Wager, Large-scale automated synthesis of human functional neuroimaging data. *Nat. Methods*. 8, 665–670 (2011).
19. G. Cumming, The New Statistics: Why and How. *Psychol. Sci*. 25, 7–29 (2014).

Fig. S1. Lower-dimensional representation of the future-thinking space in the MPFC ROI, derived from fMRI data using the 3D INDSCAL approach in Study 1. The figure illustrates projections onto Dimensions 1, 2, and 3. While Dimension 3 was difficult to interpret, a trend of negative correlation with arousal levels measured in behavioral data was observed ($\rho = -0.60$, $p = 0.066$), suggesting a modest but inconclusive association.

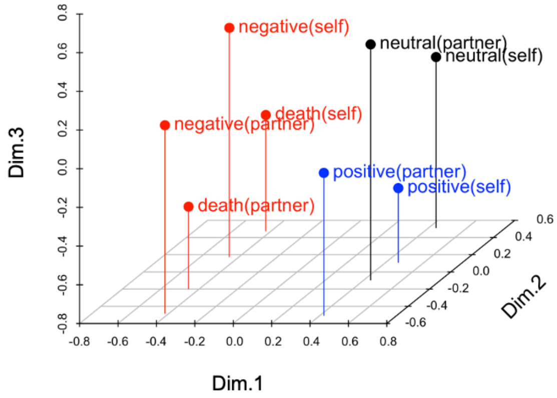

Fig. S2. Raincloud plots (**16**) depicting (a) emotional valence, (b) arousal, and (c) death relevance scores for each stimulus condition. The data were obtained from an independent group of 20 individuals who did not participate in the fMRI study (**17**). Score distributions are visualized by the density plots to the right of the box plots.

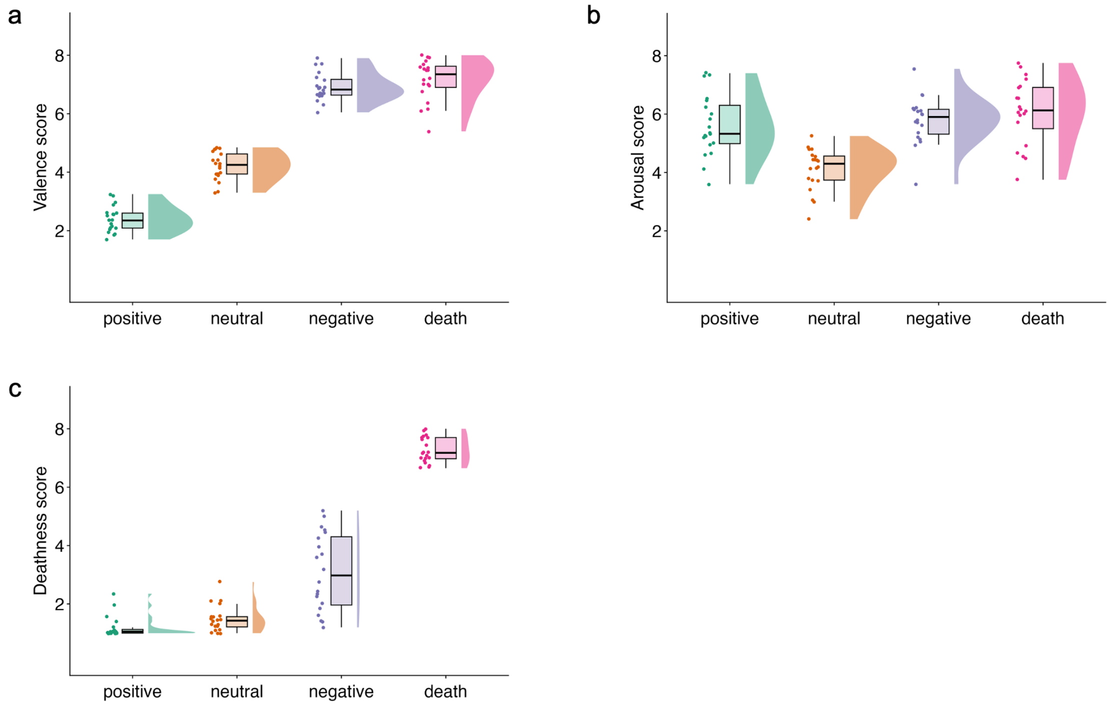

Fig. S3. Meta-analysis-derived maps illustrating brain regions preferentially associated with the prespecified search term "default mode" (**18**). All regions of interest are visualized using the xjView toolbox (http://www.alivelearn.net/xjview).

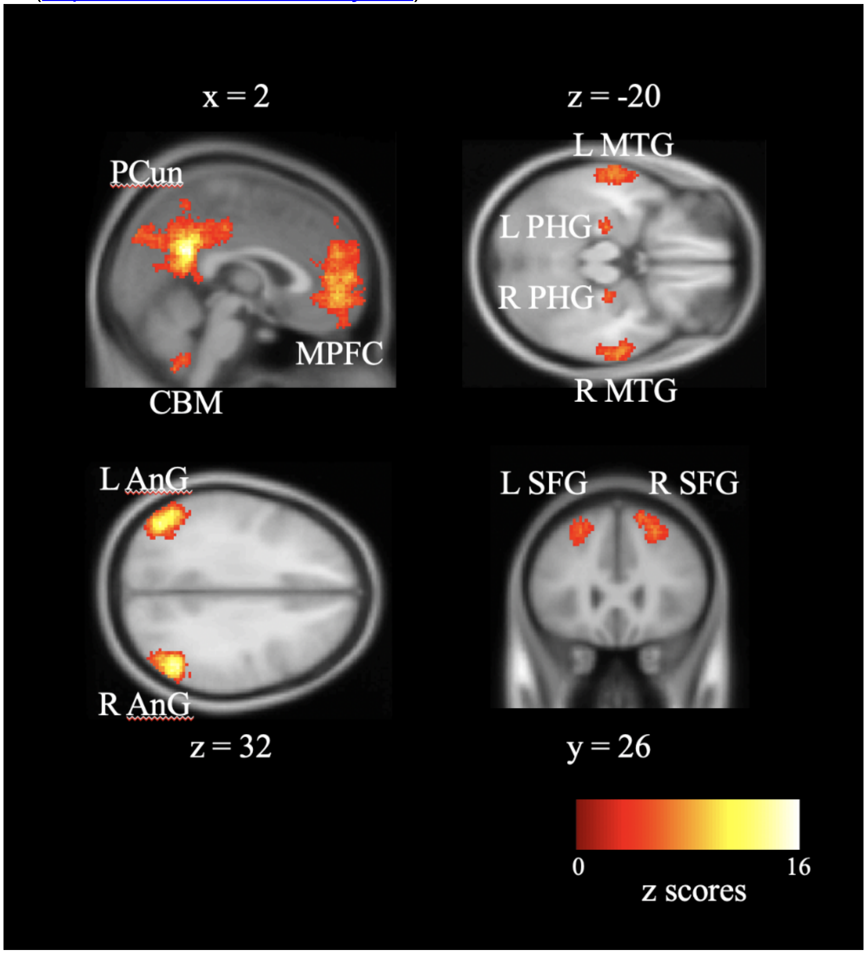

Abbreviations: CBM, cerebellum; L AnG, left angular gyrus; R AnG, right angular gyrus; L PHG, left parahippocampal gyrus; R PHG, right parahippocampal gyrus; L MTG, left middle temporal gyrus; R MTG, right middle temporal gyrus; MPFC, medial prefrontal cortex; PCun, precuneus; L SFG, left superior frontal gyrus; R SFG, right superior frontal gyrus.

Fig. S4. Behavioral similarity matrices calculated using optimism scores. In the NN model (left), individuals with more similar optimism scores are expected to exhibit increased similarity, regardless of their absolute position on the scale, which was determined by calculating the magnitude of the difference between the optimism scores of each participant pair. In contrast, the AnnaK model (right) assumes that only pairs of two highly optimistic individuals exhibit strong similarity, which was determined by computing the mean optimism score of each participant pair. The resulting matrix was rescaled to a range of 0 to 1 and then subtracted from 1 to generate the dissimilarity matrix.

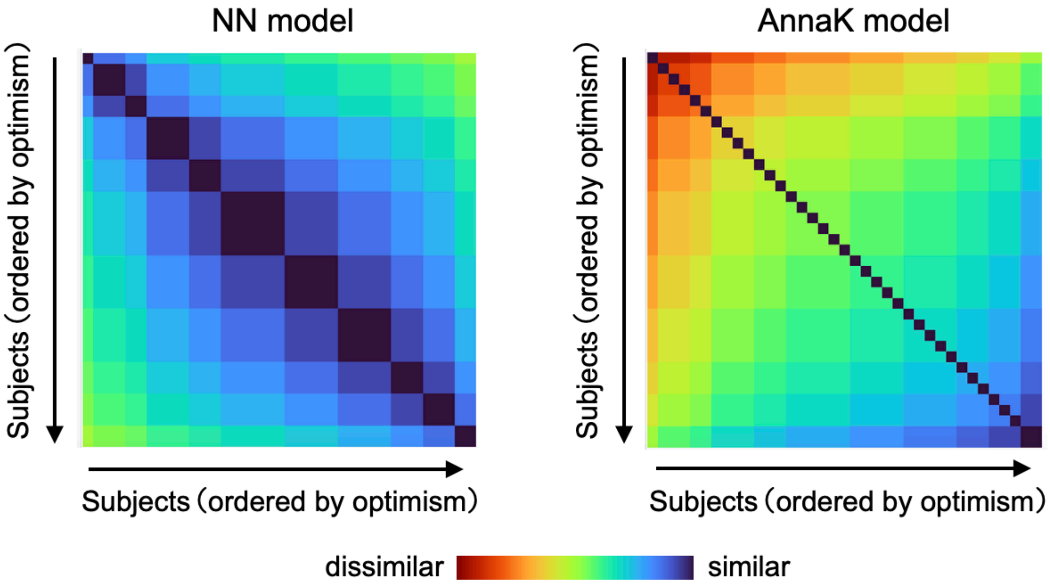

Fig. S5. (a) Distribution of LOT-R scores in Study 1 and (b) Study 2. The red dashed line represents the median score in each study.

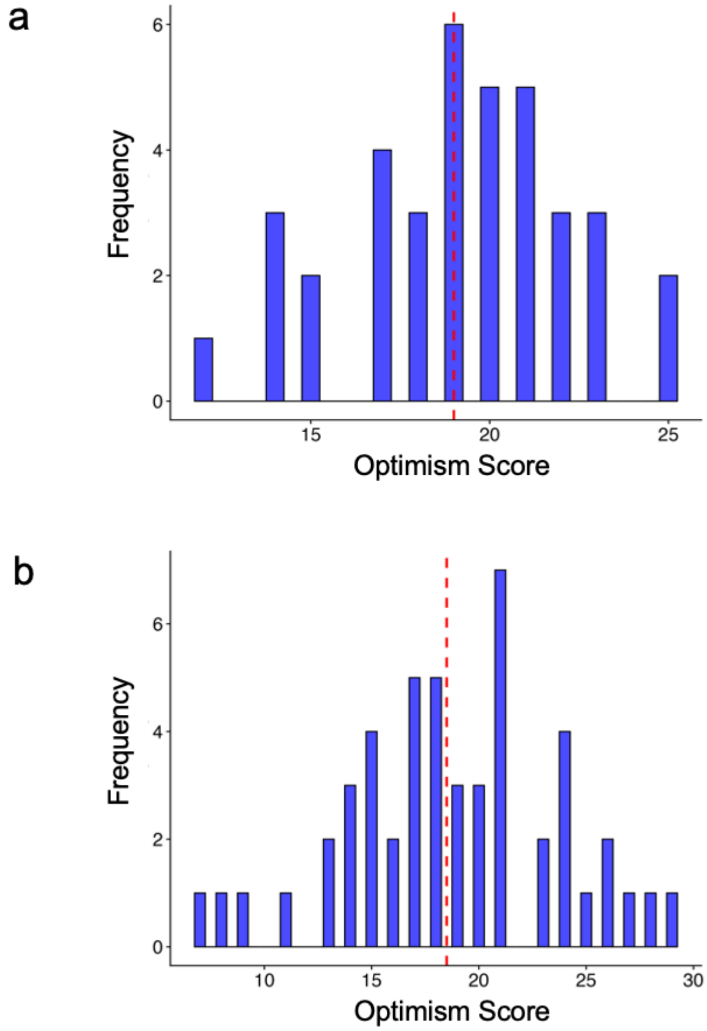

Fig. S6. Original and modified AnnaK models for behavioral similarity matrices based on optimism scores. In the original AnnaK model (left), the similarity between pairs was determined by the mean optimism score, which was rescaled and transformed into a dissimilarity matrix. In the modified AnnaK model (right), the calculation incorporated both the mean and absolute difference between scores, penalizing pairs with larger differences. The adjusted values were rescaled and converted into a dissimilarity matrix.

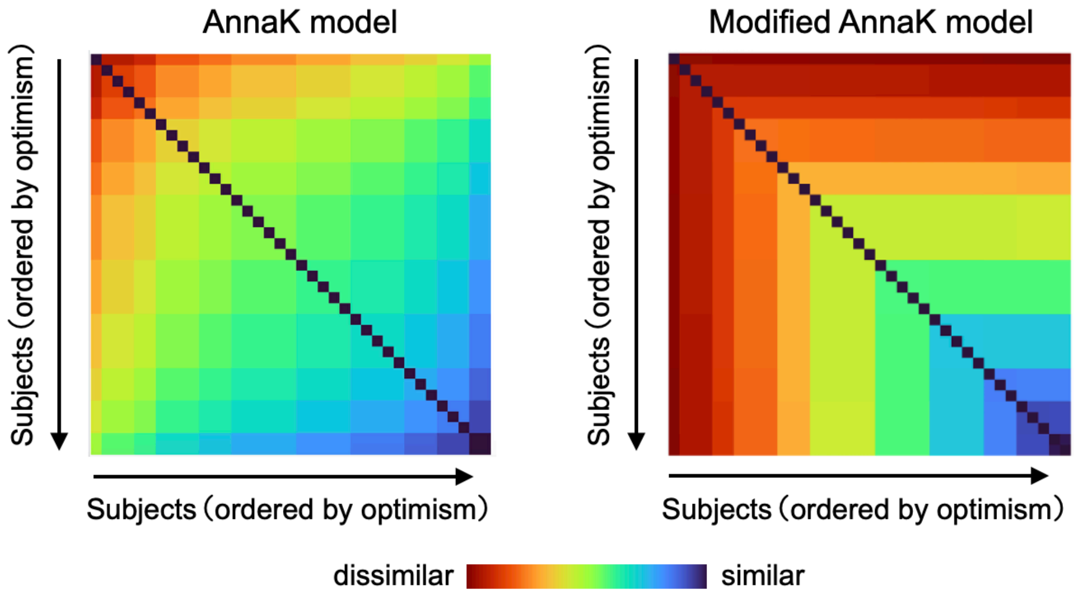

Fig. S7. Lower-dimensional representation of future-thinking space in the (a) cerebellum, (b) left angular gyrus, (c) right angular gyrus, (d) left parahippocampal gyrus, (e) right parahippocampal gyrus, (f) left middle temporal gyrus, (g) right middle temporal gyrus, (h) precuneus, (i) left superior frontal gyrus, and (j) right superior frontal gyrus, derived using the INDSCAL approach in Study 1. Colors represent different emotional valence categories.

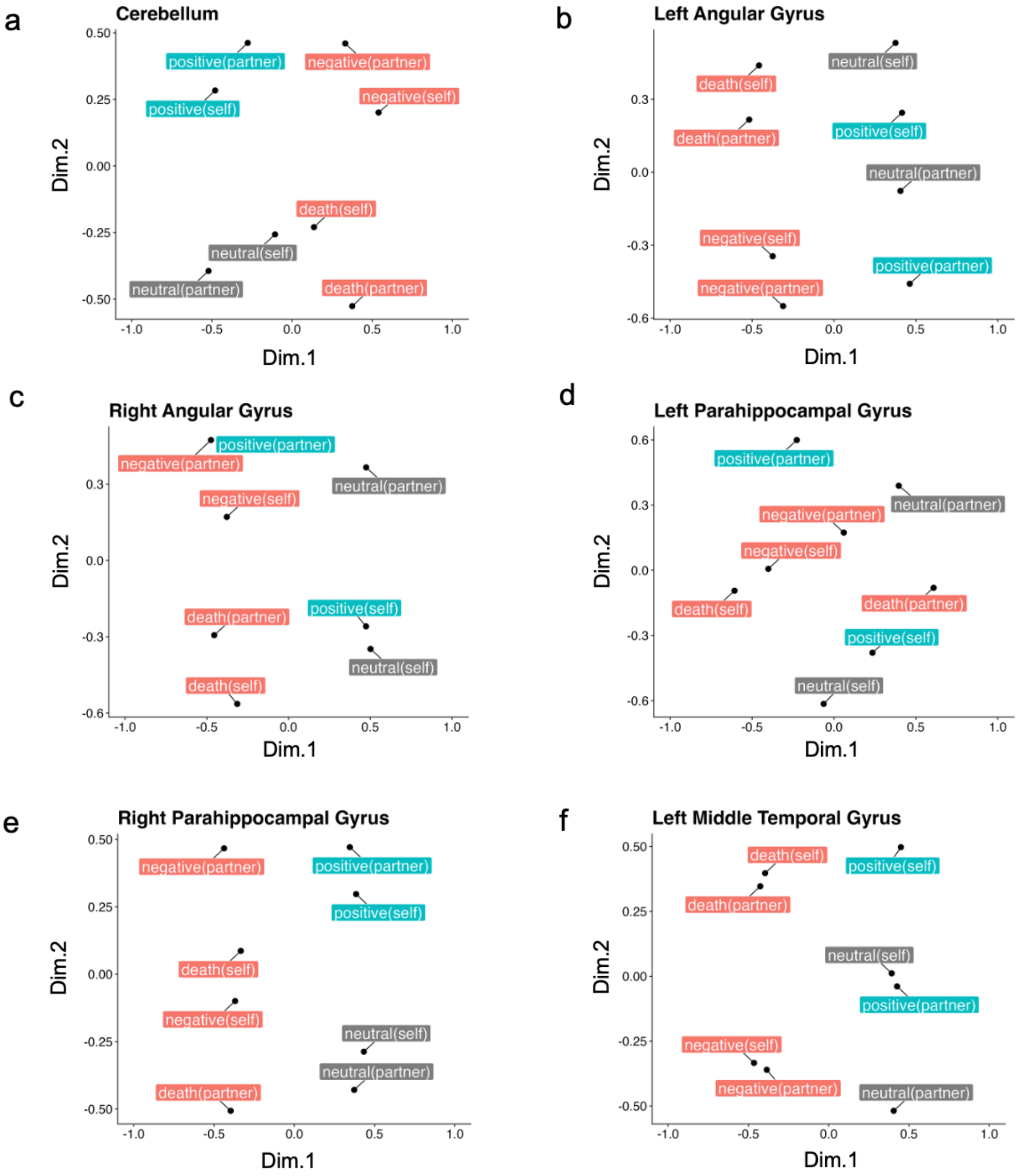

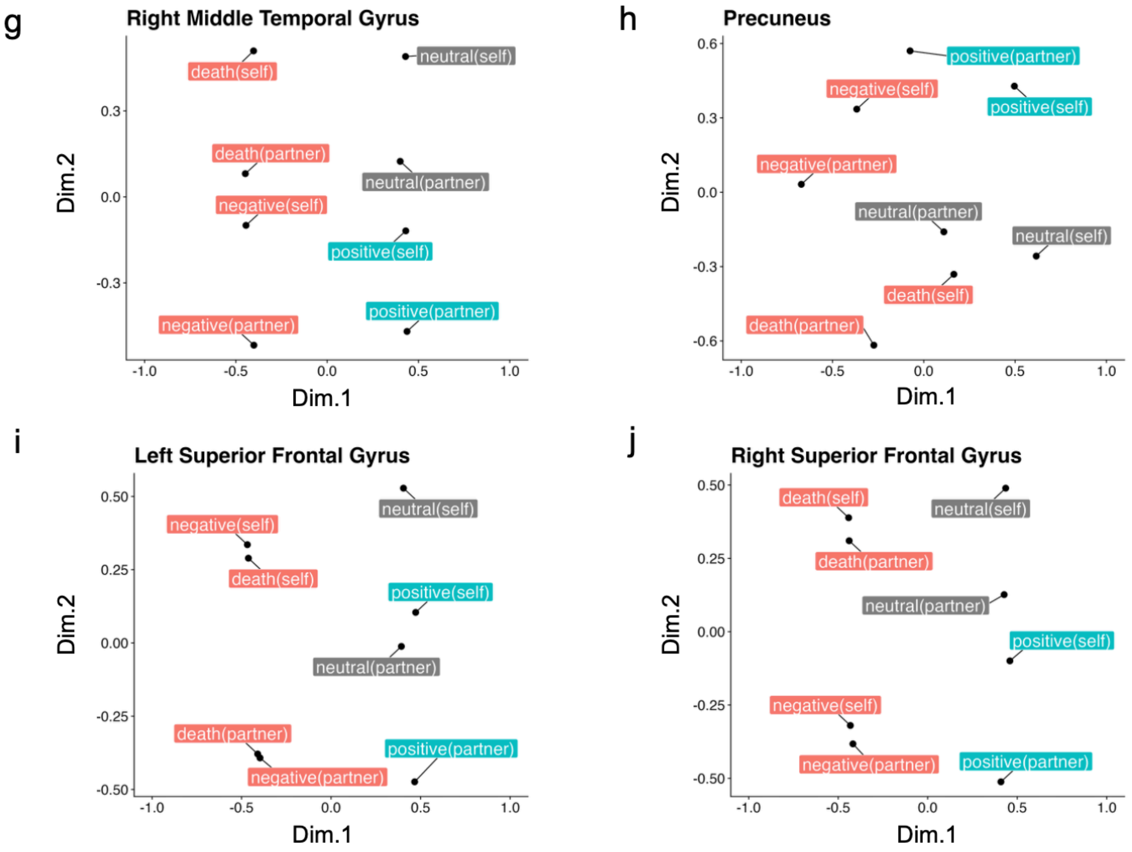

g
Right Middle Temporal Gyrus
death(self)
neutral(self)
death(partner)
neutral(partner)
negative(self)
positive(self)
negative(partner)
positive(partner)
Dim.2
Dim.1
h
Precuneus
positive(partner)
negative(self)
positive(self)
negative(partner)
neutral(partner)
neutral(self)
death(self)
death(partner)
Dim.2
Dim.1
i
Left Superior Frontal Gyrus
neutral(self)
negative(self)
death(self)
positive(self)
neutral(partner)
death(partner)
positive(partner)
negative(partner)
Dim.2
Dim.1
j
Right Superior Frontal Gyrus
death(self)
neutral(self)
death(partner)
neutral(partner)
positive(self)
negative(self)
negative(partner)
positive(partner)
Dim.2
Dim.1

Fig. S8. Lower-dimensional representation of future-thinking space in the (a) cerebellum, (b) left angular gyrus, (c) right angular gyrus, (d) left parahippocampal gyrus, (e) right parahippocampal gyrus, (f) left middle temporal gyrus, (g) right middle temporal gyrus, (h) precuneus, (i) left superior frontal gyrus, and (j) right superior frontal gyrus, derived using the INDSCAL approach in Study 2. Colors represent different emotional valence categories.

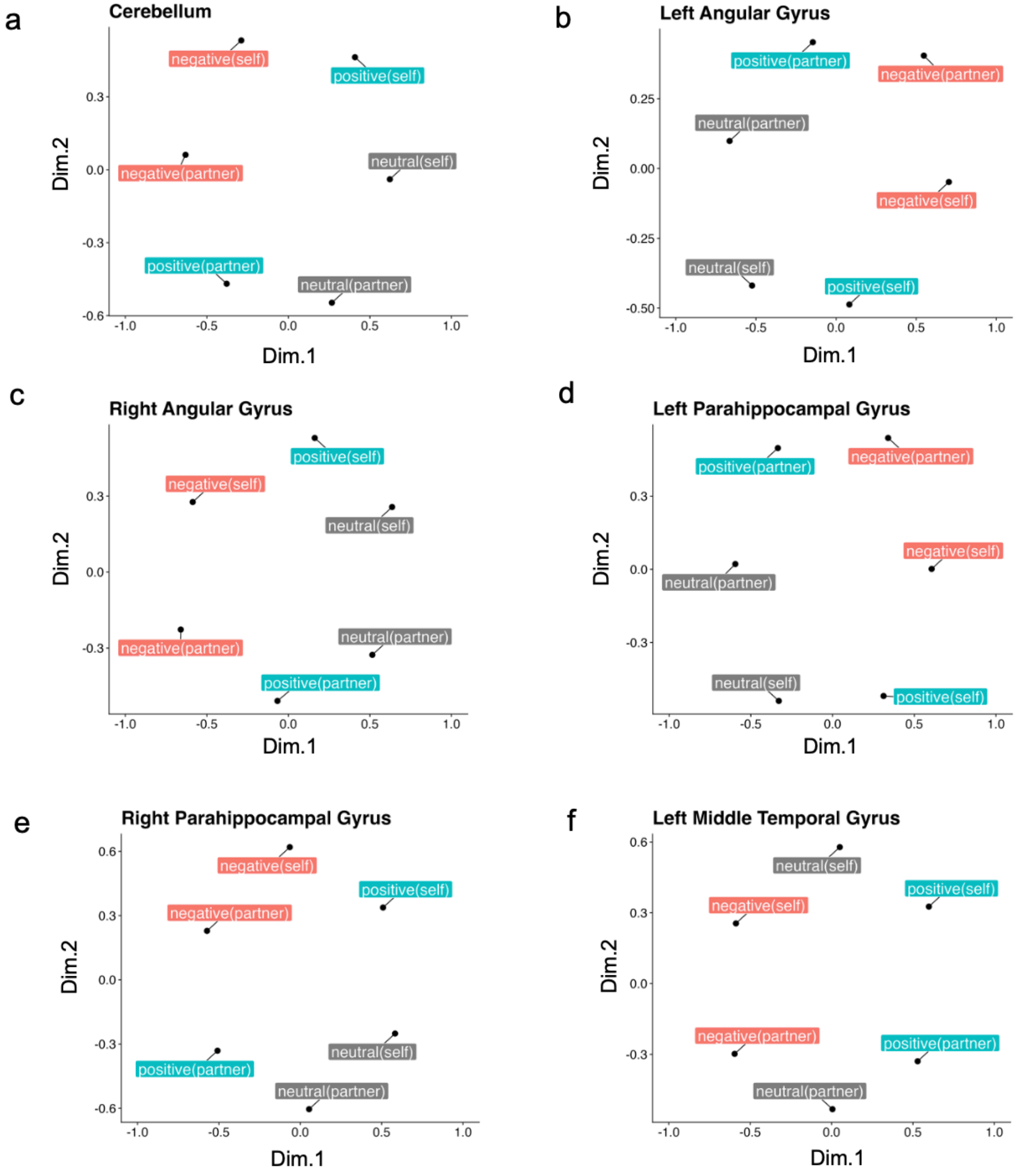

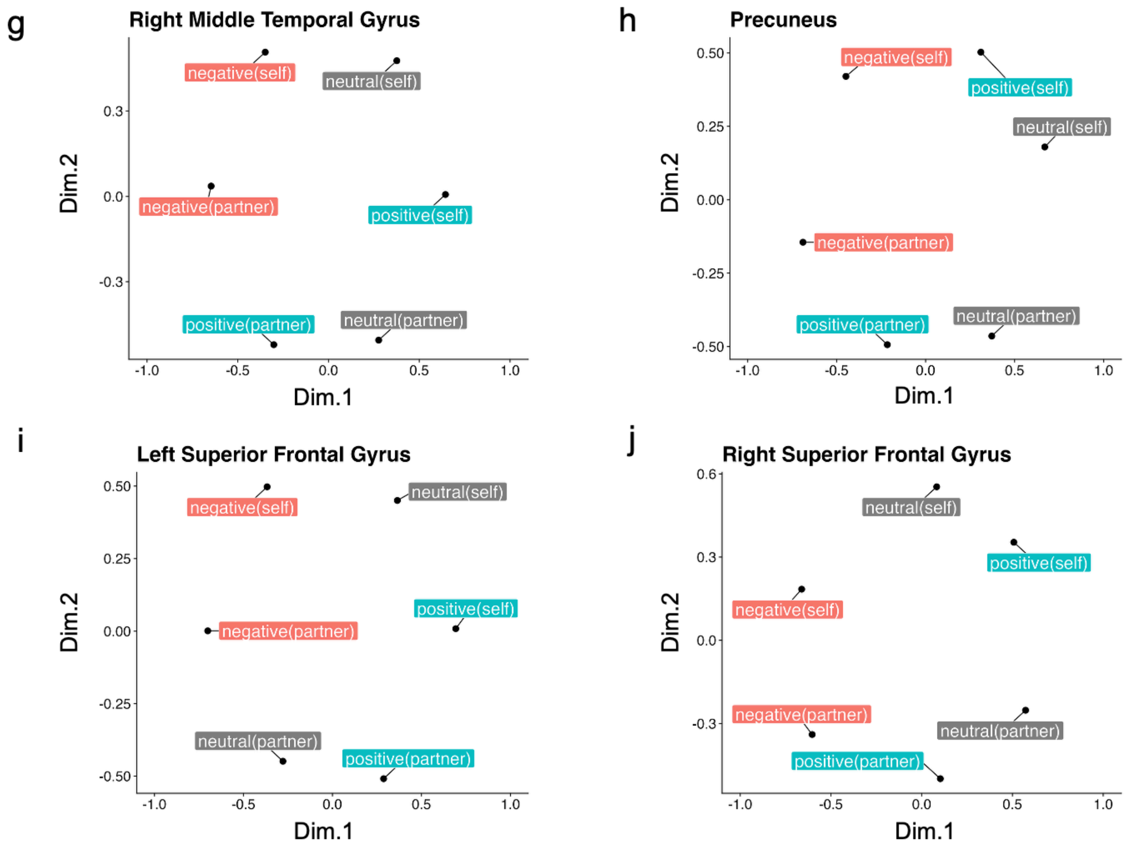
g
Right Middle Temporal Gyrus
negative(self)
neutral(self)
negative(partner)
positive(self)
positive(partner)
neutral(partner)
Dim.2
0.3
0.0
-0.3
-1.0
-0.5
0.0
0.5
1.0
Dim.1
h
Precuneus
negative(self)
positive(self)
neutral(self)
negative(partner)
positive(partner)
neutral(partner)
Dim.2
0.50
0.25
0.00
-0.25
-0.50
-1.0
-0.5
0.0
0.5
1.0
Dim.1
i
Left Superior Frontal Gyrus
negative(self)
neutral(self)
positive(self)
negative(partner)
neutral(partner)
positive(partner)
Dim.2
0.50
0.25
0.00
-0.25
-0.50
-1.0
-0.5
0.0
0.5
1.0
Dim.1
j
Right Superior Frontal Gyrus
neutral(self)
positive(self)
negative(self)
negative(partner)
neutral(partner)
positive(partner)
Dim.2
0.6
0.3
0.0
-0.3
-1.0
-0.5
0.0
0.5
1.0
Dim.1

Fig. S9. MDS of ROI similarities. Two-dimensional MDS plots based on pairwise Mantel correlation coefficients between ROIs. (a) MDS results from Study 1 and (b) MDS results from Study 2. Each point represents an ROI, with distances between points reflecting the similarity in representational structures, as determined by the correlations of their neural dissimilarity matrices. Abbreviations: CBM, cerebellum; L AnG, left angular gyrus; R AnG, right angular gyrus; L PHG, left parahippocampal gyrus; R PHG, right parahippocampal gyrus; L MTG, left middle temporal gyrus; R MTG, right middle temporal gyrus; MPFC, medial prefrontal cortex; PCun, precuneus; L SFG, left superior frontal gyrus; R SFG, right superior frontal gyrus.

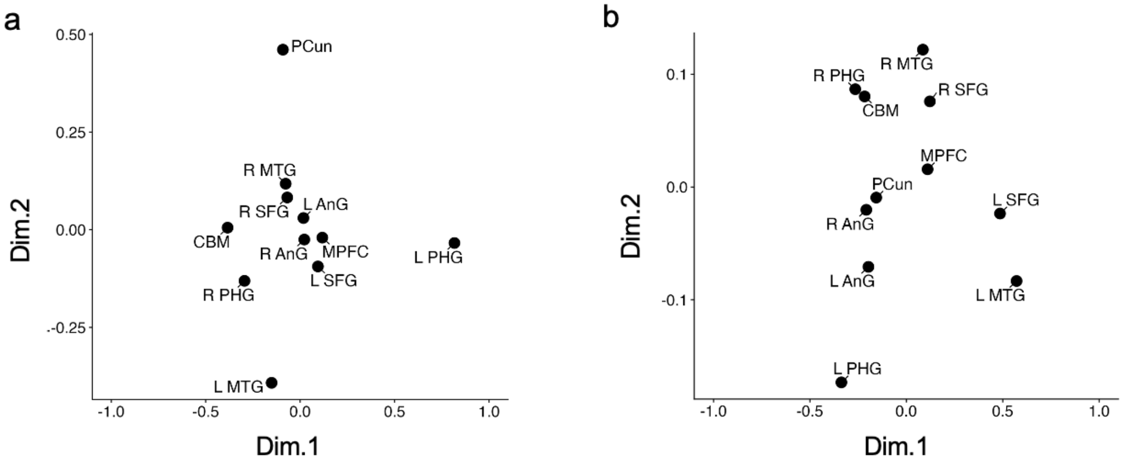

Fig. S10. Raincloud plots showing individual signal changes in the MPFC for each condition in the fMRI task. Results are displayed for (a) Study 1 and (b) Study 2, with data separated by self- and partner-referential conditions across emotion categories: death, negative, neutral, and positive. Each plot illustrates individual data points, box plots representing group medians and interquartile ranges, and distributions visualized to the right of the box plots.

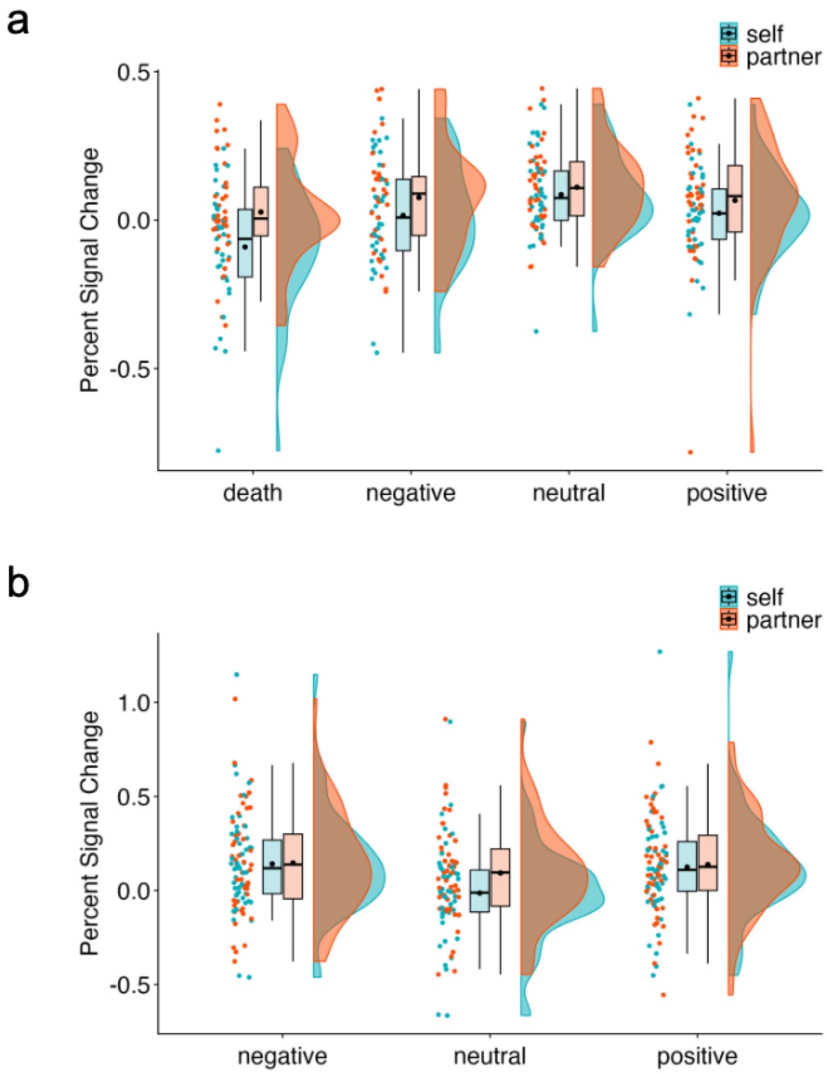

Fig. S11. Lower-dimensional representation of future-thinking space in the (a) cerebellum, (b) left angular gyrus, (c) right angular gyrus, (d) left parahippocampal gyrus, (e) right parahippocampal gyrus, (f) left middle temporal gyrus, (g) right middle temporal gyrus, (h) medial prefrontal cortex, (i) precuneus, (j) left superior frontal gyrus, and (k) right superior frontal gyrus, using the univariate-based INDSCAL approach in Study 1. Colors indicate emotional valence categories.

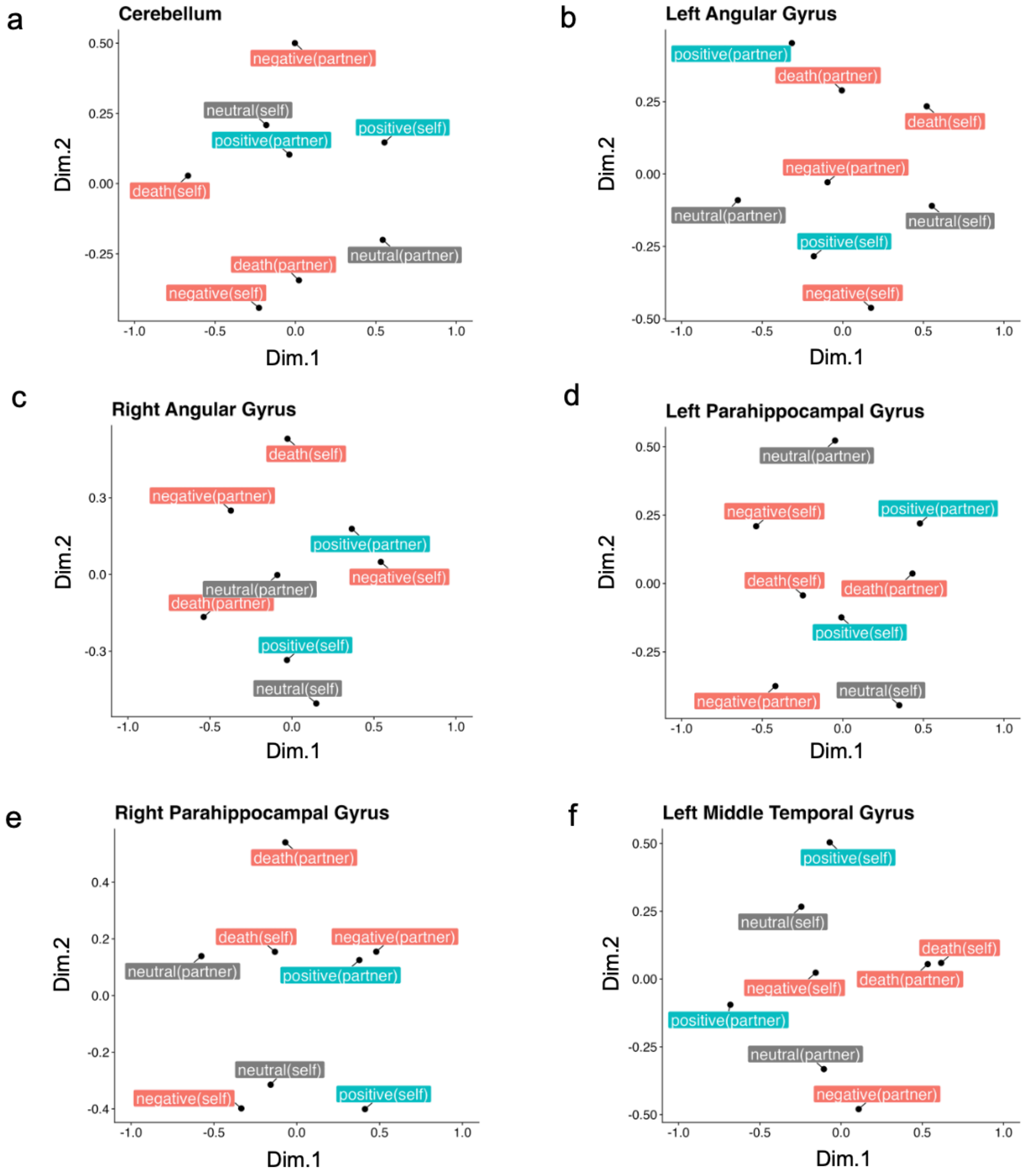

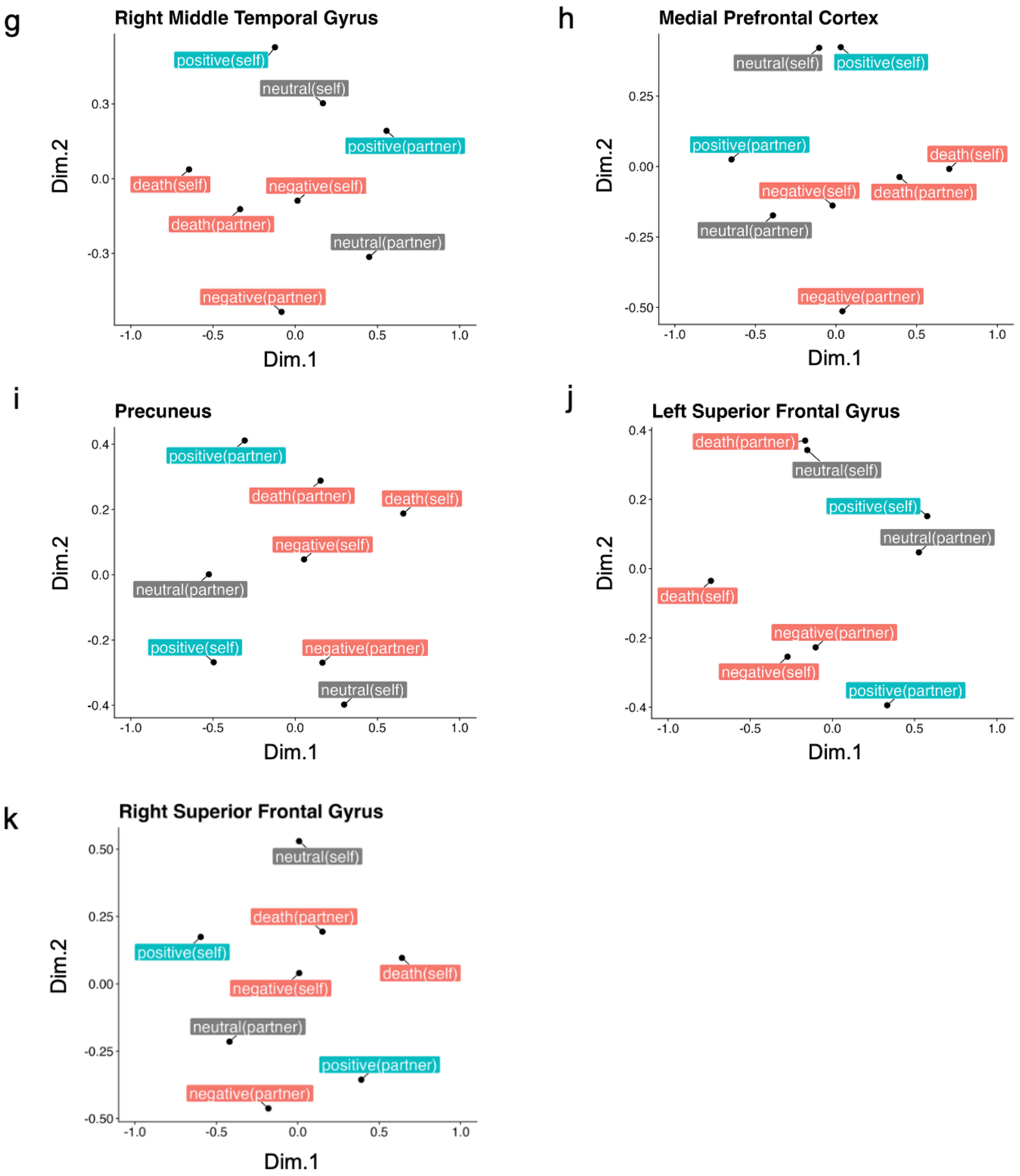
g
Right Middle Temporal Gyrus
positive(self)
neutral(self)
positive(partner)
death(self)
negative(self)
death(partner)
neutral(partner)
negative(partner)
Dim.2
Dim.1
h
Medial Prefrontal Cortex
neutral(self)
positive(self)
positive(partner)
death(self)
negative(self)
death(partner)
neutral(partner)
negative(partner)
Dim.2
Dim.1
i
Precuneus
positive(partner)
death(partner)
death(self)
negative(self)
neutral(partner)
positive(self)
negative(partner)
neutral(self)
Dim.2
Dim.1
j
Left Superior Frontal Gyrus
death(partner)
neutral(self)
positive(self)
neutral(partner)
death(self)
negative(partner)
negative(self)
positive(partner)
Dim.2
Dim.1
k
Right Superior Frontal Gyrus
neutral(self)
death(partner)
positive(self)
death(self)
negative(self)
neutral(partner)
positive(partner)
negative(partner)
Dim.2
Dim.1

Fig. S12. Lower-dimensional representation of future-thinking space in the (a) cerebellum, (b) left angular gyrus, (c) right angular gyrus, (d) left parahippocampal gyrus, (e) right parahippocampal gyrus, (f) left middle temporal gyrus, (g) right middle temporal gyrus, (h) medial prefrontal cortex, (i) precuneus, (j) left superior frontal gyrus, and (k) right superior frontal gyrus, derived using the univariate-based INDSCAL approach in Study 2. Colors represent different emotional valence categories.

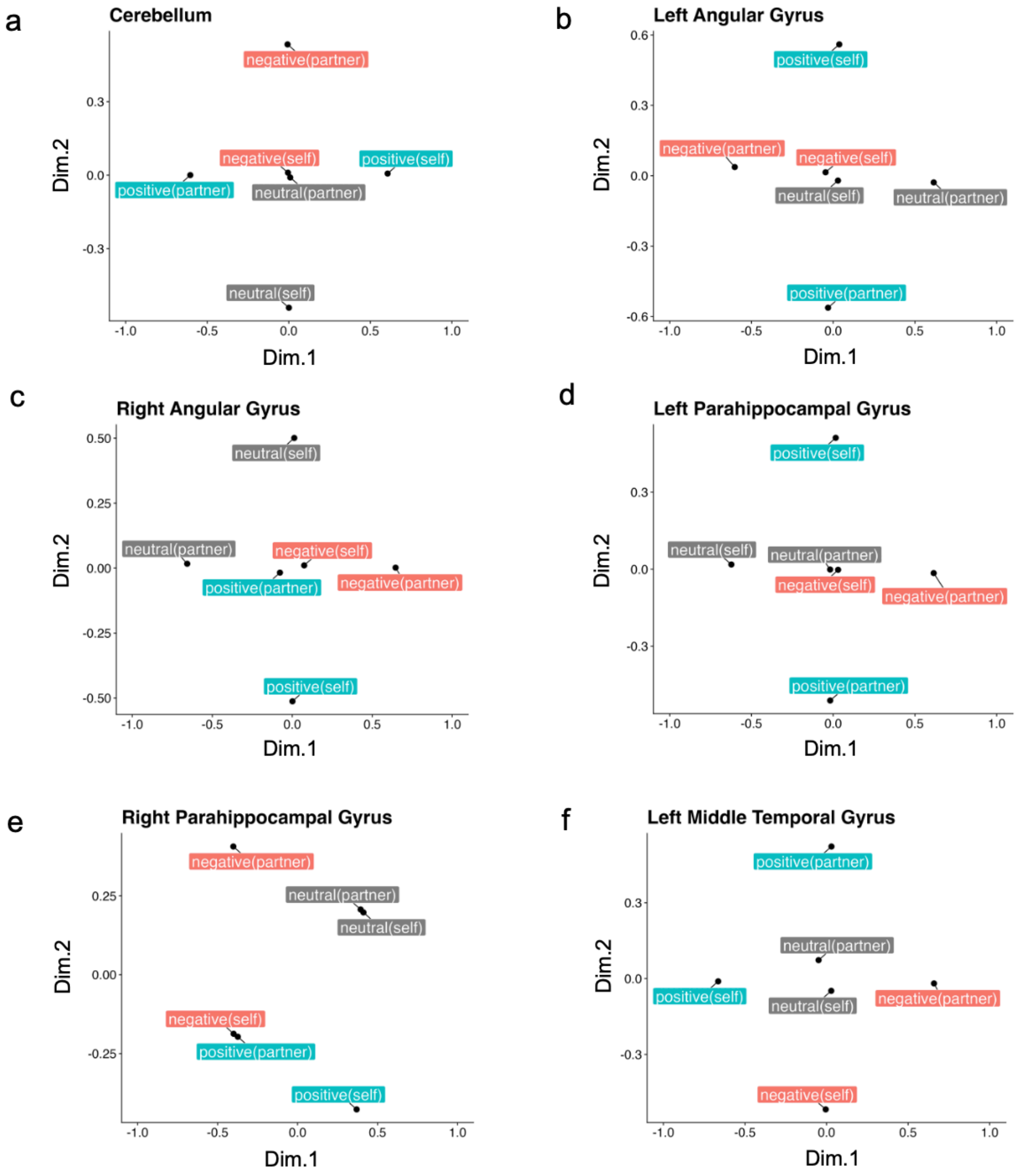

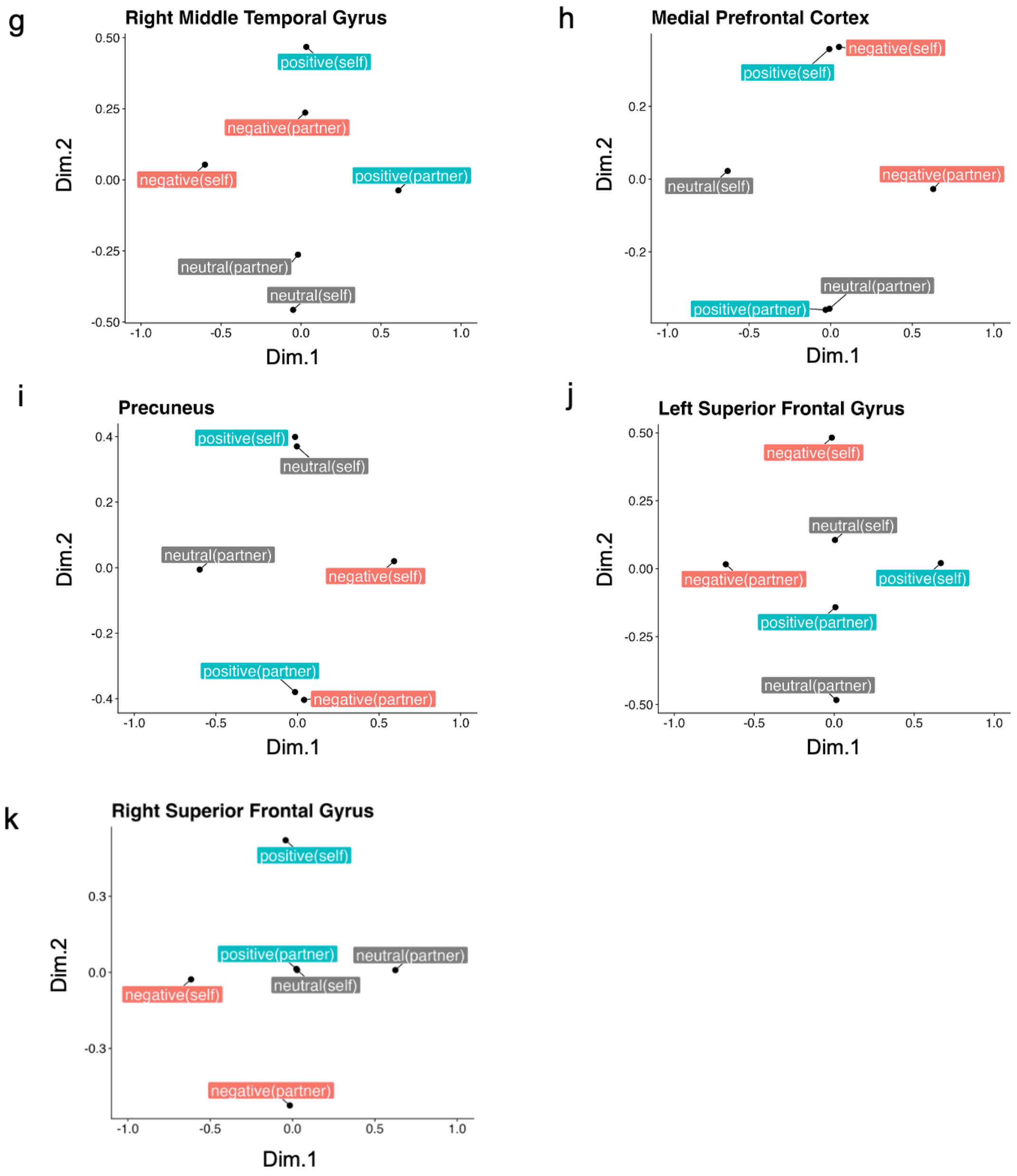
g
Right Middle Temporal Gyrus
positive(self)
negative(partner)
negative(self)
positive(partner)
neutral(partner)
neutral(self)
Dim.2
Dim.1
0.50
0.25
0.00
-0.25
-0.50
-1.0
-0.5
0.0
0.5
1.0
h
Medial Prefrontal Cortex
negative(self)
positive(self)
neutral(self)
negative(partner)
neutral(partner)
positive(partner)
Dim.2
Dim.1
0.2
0.0
-0.2
i
Precuneus
positive(self)
neutral(self)
neutral(partner)
negative(self)
positive(partner)
negative(partner)
Dim.2
Dim.1
0.4
0.2
0.0
-0.2
-0.4
j
Left Superior Frontal Gyrus
negative(self)
neutral(self)
negative(partner)
positive(self)
positive(partner)
neutral(partner)
Dim.2
Dim.1
k
Right Superior Frontal Gyrus
positive(self)
positive(partner)
neutral(partner)
negative(self)
neutral(self)
negative(partner)
Dim.2
Dim.1
0.3
0.0
-0.3

Fig. S13. MDS visualization of participant similarity on the basis of MPFC neural patterns. (a) Results obtained in Study 1. (b) Results obtained in Study 2. Each dot represents a participant, and the dots are colored according to their INDSCAL Dimension 1 weights. The crosses indicate the densest regions, as identified via Gaussian kernel density estimation. Note that Dimension 1 on the x-axis and Dimension 2 on the y-axis represent the dimensions of the MDS space based on the MVPA-based matrix, and are different from the dimensions in the INDSCAL results.

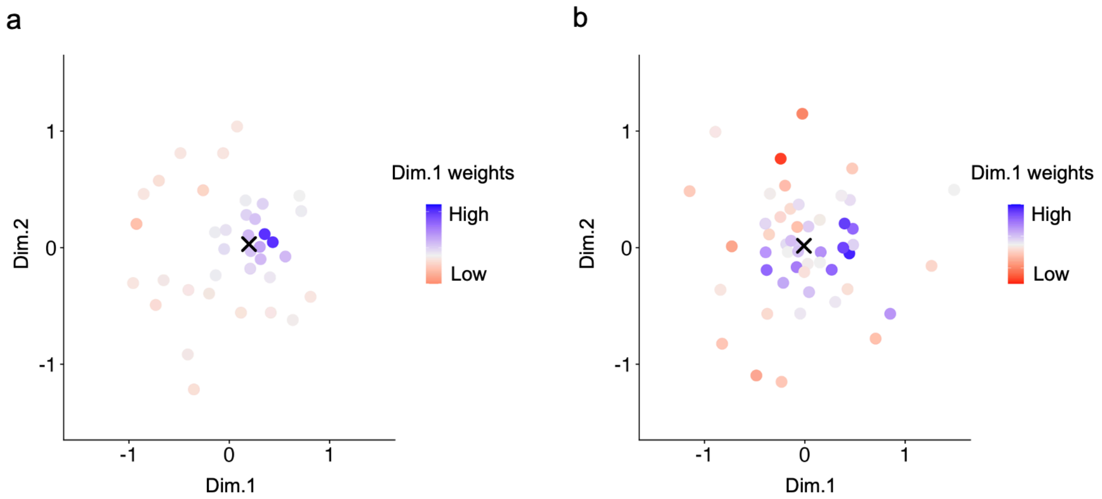

Fig. S14. (a) Associations between intersubject dissimilarity and optimism in Study 1, which are identified on the basis of cross-run correlation distances. This matrix pertains to an ROI in the MPFC, and rows and columns are arranged in ascending order of optimism. The top-left corner represents the pair with the lowest optimism scores, whereas the bottom-right corner corresponds to the pair with the highest optimism scores. Warm colors represent high dissimilarity values, whereas cool colors indicate low dissimilarity values. (b) MDS visualization for Study 1, which is identified on the basis of cross-run correlation distances. Nonmetric MDS was used to represent the similarity among participants on the basis of their neural patterns in the MPFC. Each dot represents a participant, and colors reflect optimism scores. Gaussian kernel density estimation was used to identify the region with the highest density of points, which is marked with a cross. (c) Associations between intersubject dissimilarity and optimism in Study 2, which were identified on the basis of cross-run correlation distances. The matrix was constructed via the same procedure that was employed in Study 1. (d) MDS results for Study 2, which were identified on the basis of cross-run correlation distances. MDS was conducted via the same procedure that was employed in Study 1, and the densest region in the MDS space was identified and marked with a cross. Note that Dimension 1 and Dimension 2 presented here are different from Dimension 1 and Dimension 2 in the INDSCAL results.

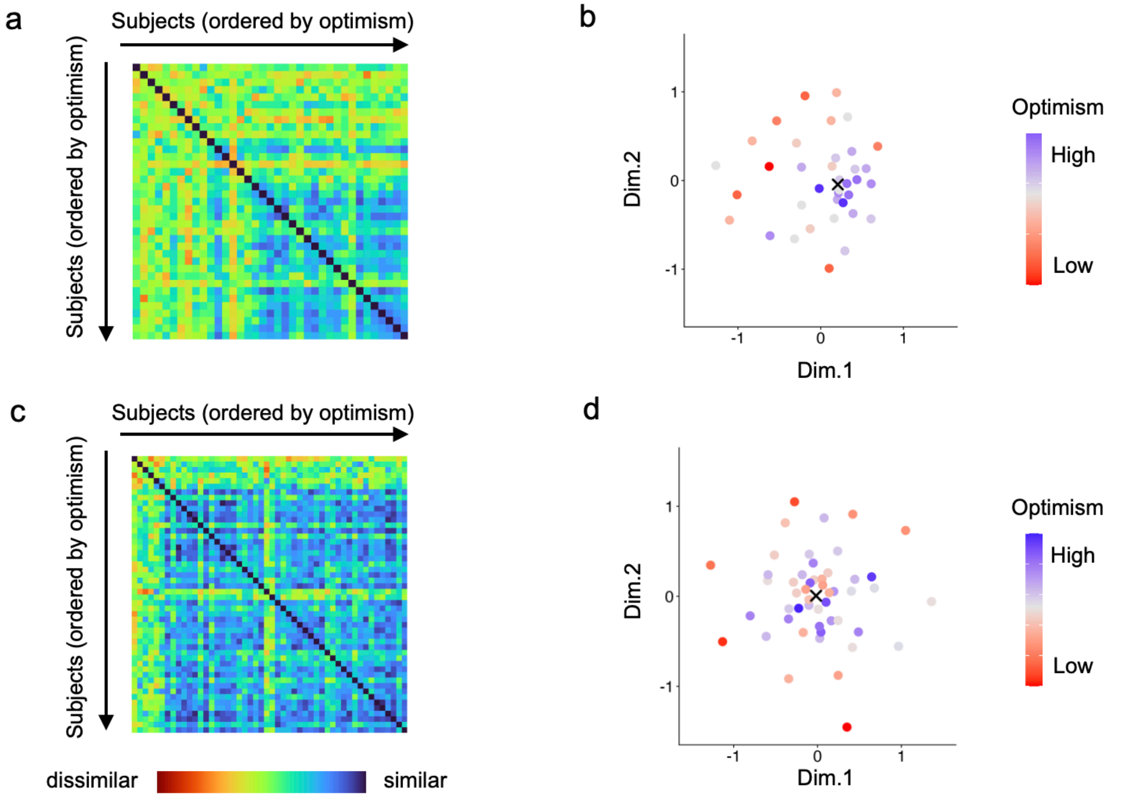

Fig. S15. (a) Lower-dimensional representation of the future-thinking neural space in the MPFC ROI, which is derived from cross-run correlation distances obtained via the INDSCAL approach (Study 1). Each color represents an emotional valence condition. (b) Individual subject weights from the INDSCAL analysis on the basis of cross-run correlation distances (Study 1), thus reflecting the importance of the two dimensions for each participant. Each dot represents a participant, and colors reflect optimism scores. (c) Lower-dimensional representation of neural patterns in Study 2, which is derived from cross-run correlation distances obtained via the same INDSCAL procedures that were employed in Study 1. (d) Individual subject weights in Study 2, which were obtained via the same INDSCAL approach pertaining to cross-run correlation distances that was employed in Study 1.

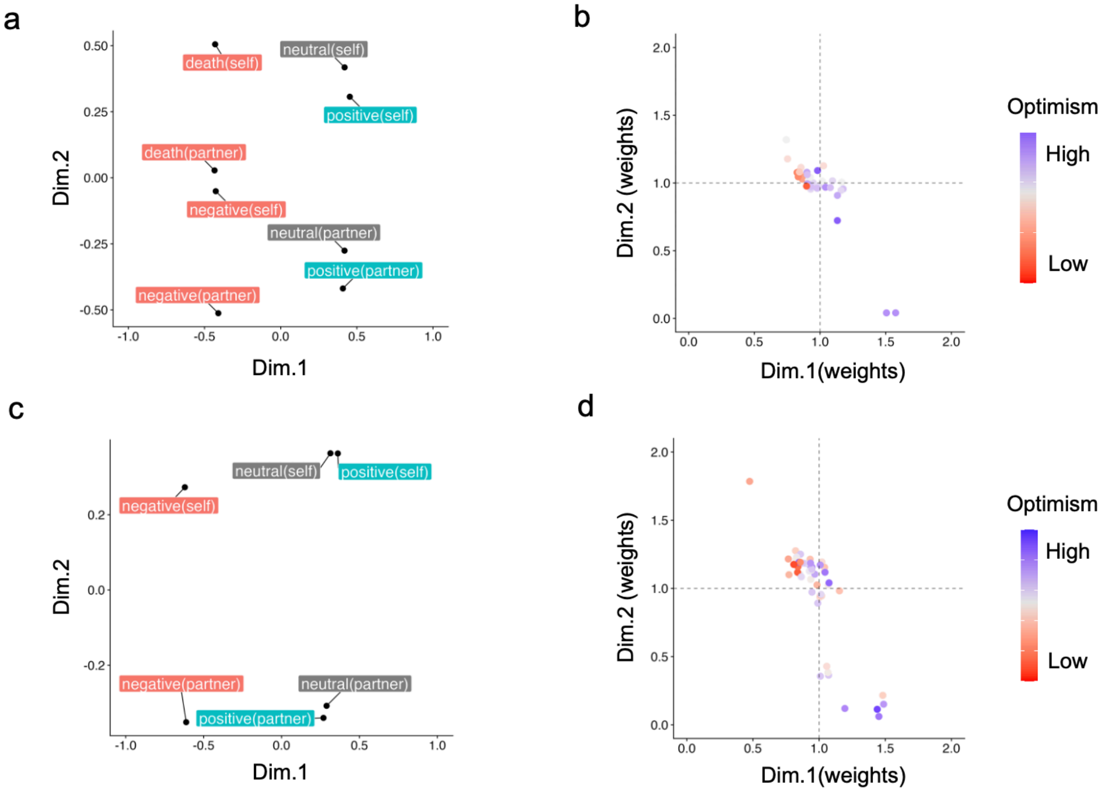

Fig. S16. Raincloud plots showing individual (a) emotional valence, (b) arousal, (c) death relevance, (d) vividness, (e) projection, (f) temporal distance, and (g) self-relevance scores for each condition in the behavioral task. The data were obtained from an independent group of 20 married individuals who did not participate in the fMRI study. The shapes visualize score distributions to the right of the box plots.

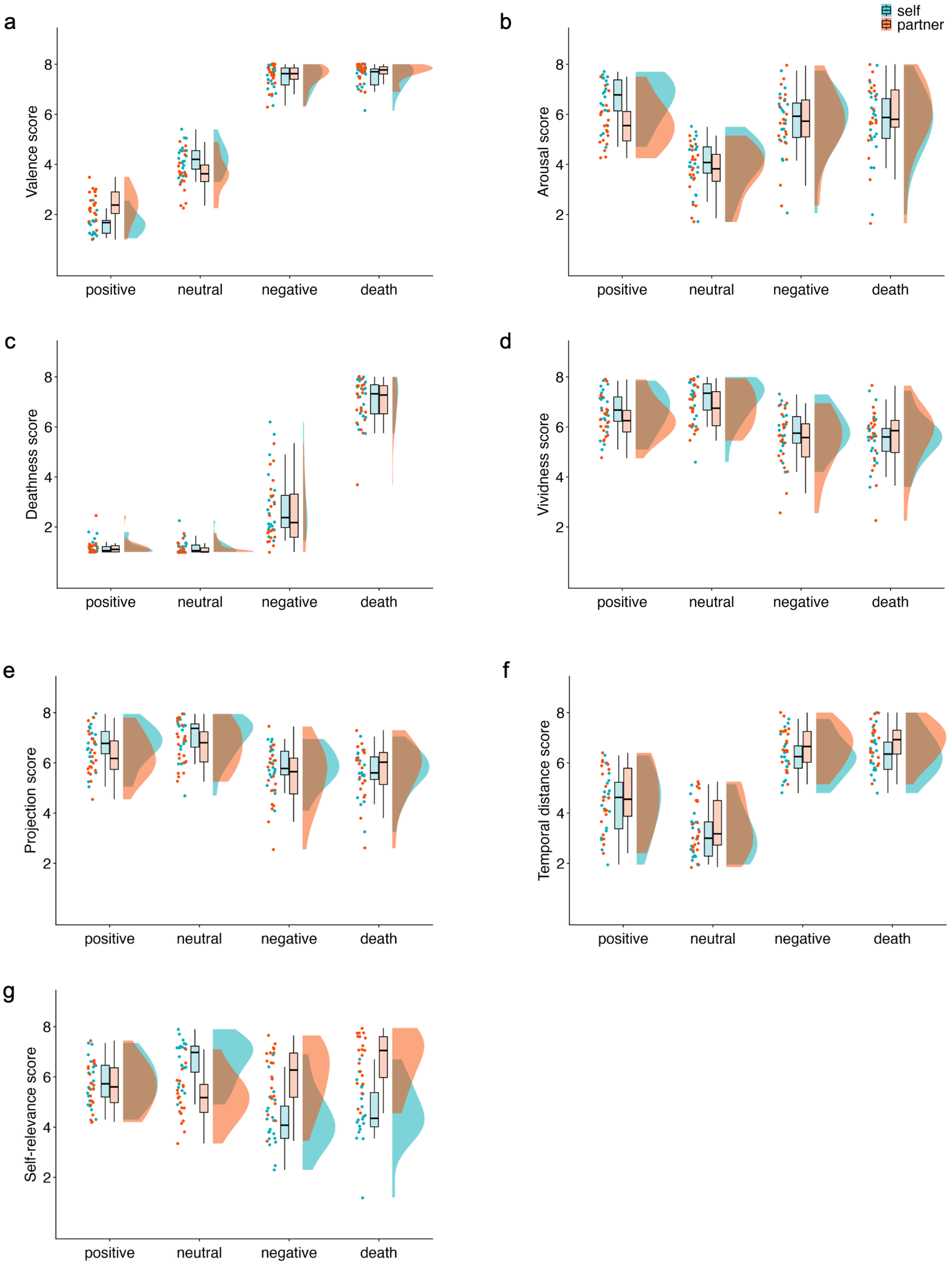

Table S1. Correlations between the Neural RDMs and Candidate Model RDMs (AnnaK and NN models) across ROIs

Region	ρ (Study 1)	Corrected *p value* (Study 1)	ρ (Study 2)	Corrected *p value* (Study 2)
**AnnaK**				
Cerebellum	0.001	1.000	0.156	0.042
Lt. angular gyrus	0.302	0.057	0.221	0.183
Rt. angular gyrus	0.148	1.000	0.284	0.036
Lt. parahippocampal gyrus	0.133	0.003	0.071	0.133
Rt. parahippocampal gyrus	0.017	1.000	0.057	0.936
Lt. middle temporal gyrus	0.342	0.012	0.233	0.034
Rt. middle temporal gyrus	0.209	0.220	0.172	0.132
Medial prefrontal cortex	0.419	0.001	0.377	0.003
Precuneus	0.269	0.176	0.292	0.024
Lt. superior frontal gyrus	0.283	0.056	0.208	0.113
Rt. superior frontal gyrus	0.318	0.009	0.308	0.016
**NN**				
Cerebellum	-0.007	1.000	0.025	1.000
Lt. angular gyrus	0.105	1.000	0.077	1.000
Rt. angular gyrus	0.063	1.000	0.071	1.000
Lt. parahippocampal gyrus	0.103	1.000	0.086	0.067
Rt. parahippocampal gyrus	-0.075	1.000	0.015	1.000
Lt. middle temporal gyrus	0.136	0.532	0.056	1.000
Rt. middle temporal gyrus	0.008	1.000	0.031	1.000
Medial prefrontal cortex	0.167	0.250	0.169	0.156
Precuneus	0.057	1.000	0.129	0.405
Lt. superior frontal gyrus	-0.032	1.000	0.044	1.000
Rt. superior frontal gyrus	0.060	1.000	0.117	0.601

Lt, left; Rt, right.

Table S2. Correlations and Descriptive Statistics for Optimism and Control Variables in Study 2

Variable	*M*	*SD*	1	2	3	4
1. Age	34.04	4.34				
2. Optimism	18.74	4.99	-.09<br>[-.36, .19]			
3. SES	5.30	1.67	-.10<br>[-.37, .19]	.07<br>[-.21, .34]		
4. Education level	4.86	1.63	-.07<br>[-.35, .21]	.24<br>[-.04, .49]	.40**<br>[.14, .61]	
5. Nonverbal intelligence	33.16	3.93	-.08<br>[-.35, .20]	.07<br>[-.21, .35]	.17<br>[-.12, .43]	.09<br>[-.19, .36]

*Note. M and SD represent the mean and standard deviation, respectively. Values in square brackets indicate the 95% confidence interval for each correlation. The confidence interval reflects the plausible range of population correlations that could have produced the observed sample correlation (19). p < .05 is indicated by *, and p < .01 is indicated by **.*

Table S3. Results Examining the Relationship between Optimism and Neural Representational Similarity in Study 1

Region	β	SE	*p value* (corrected)
**$ISC_{\{high, high\}}$ > $ISC_{\{low, low\}}$**			
Medial prefrontal cortex	1.070	0.313	< 0.001
Lt. parahippocampal gyrus	0.333	0.113	0.001
Lt. middle temporal gyrus	0.825	0.319	0.009
Rt. superior frontal gyrus	0.826	0.289	0.002
**$ISC_{\{high, high\}}$ > $ISC_{\{high, low\}}$**			
Medial prefrontal cortex	0.779	0.166	< 0.001
Lt. parahippocampal gyrus	0.321	0.097	< 0.001
Lt. middle temporal gyrus	0.577	0.170	< 0.001
Rt. superior frontal gyrus	0.557	0.157	< 0.001
**$ISC_{\{high, low\}}$ > $ISC_{\{low, low\}}$**			
No suprathreshold regions			

Lt, left; Rt, right; ISC, Intersubject correlations.

Table S4. Results Examining the Relationship between Optimism and Neural Representational Similarity in Study 2

Region	β	SE	*p value* (corrected)
$\mathbf{ISC_{\{high,\ high\}} > ISC_{\{low,\ low\}}}$			
Medial prefrontal cortex	0.770	0.312	0.017
Rt. superior frontal gyrus	0.693	0.292	0.027
$\mathbf{ISC_{\{high,\ high\}} > ISC_{\{high,\ low\}}}$			
Medial prefrontal cortex	0.402	0.160	0.013
Cerebellum	0.251	0.099	0.011
Rt. superior frontal gyrus	0.362	0.151	0.024
$\mathbf{ISC_{\{high,\ low\}} > ISC_{\{low,\ low\}}}$			
Medial prefrontal cortex	0.368	0.160	0.039

Lt, left; Rt, right; ISC, Intersubject correlations.
